# Mapping CSC‐Mediated Ovarian Cancer Chemoresistance via CXCR4‐PET to Guide Precision Cisplatin Re‐Sensitization Therapy

**DOI:** 10.1002/advs.202521279

**Published:** 2026-02-03

**Authors:** Lixia Feng, Simei Zhao, Zheng Wei, Wenwen Wang, Feiquan Ying, Lin Huang, Mengna Zhu, Mengqing Chen, Qiang Yang, Si Sun, Dawei Jiang, Lingling Gao, Jing Cai

**Affiliations:** ^1^ Department of Obstetrics and Gynecology Union Hospital Tongji Medical College Huazhong University of Science and Technology Wuhan China; ^2^ Department of Nuclear Medicine Union Hospital Tongji Medical College Huazhong University of Science and Technology Wuhan China; ^3^ Hubei Key Laboratory of Molecular Imaging Wuhan China; ^4^ Key Laboratory of Biological Targeted Therapy The Ministry of Education Wuhan China; ^5^ Department of Obstetrics and Gynecology Third Hospital of Shanxi Medical University Shanxi Bethune Hospital Shanxi Academy of Medical Sciences Tongji Shanxi Hospital Taiyuan China; ^6^ Department of Obstetrics and Gynecology Zhongnan Hospital of Wuhan University Wuhan China

**Keywords:** cancer stem cells, chemoresistance, CXC motif chemokine receptor 4, ovarian cancer, positron emission tomography/computed tomography

## Abstract

Therapy targeting cancer stem cells (CSCs) has been proposed as a promising strategy to reduce chemoresistance and relapse risks in ovarian cancer (OC) patients. However, the lack of targetable markers impedes research progress. Here, we demonstrate that CXC motif chemokine receptor 4 (CXCR4) may be a targetable functional marker of ovarian CSCs and propose a new translational model incorporating targeted imaging and CXCR4 blockade in CXCR4^+^ tumors. Expression profile analysis of chemoresistant CSC‐like ovarian cancer cells highlighted that CXCR4 functions as a potential stemness marker. CXCR4^+^ ovarian cancer cells exhibited high self‐renewal capacity in vitro and in vivo, and an association with chemoresistance. CXCR4 inhibitor AMD3100 significantly impaired the self‐renewal ability of CSC‐like ovarian cancer cells and enhanced their sensitivity to cisplatin. CXCR4‐targeted [^68^Ga]Ga‐Pentixafor was highly specific in delineating CXCR4‐high cell line‐derived xenografts and patient‐derived xenografts (PDXs) via positron emission tomography (PET) imaging, with precise tumor‐targeting and persistent retention. A combination of AMD3100 and CDDP exerted an excellent antitumor effect in CXCR4‐high PDXs, but not in CXCR4‐low PDXs. These results suggest that CXCR4 may represent a functional CSC marker associated with chemoresistance. Moreover, [^68^Ga]Ga‐Pentixafor PET imaging can guide decision‐making for AMD3100 therapy, paving the way for further clinical translation.

## Graphical abstract

CXCR4 functions as a potential cancer stem cell‐associated marker linked to chemoresistance in ovarian cancer, and an inhibitor of CXCR4, AMD3100, can enhance the cytotoxic response to cisplatin. Based on these findings, [^68^Ga]Ga‐Pentixafor positron emission tomography enables selective visualization of CXCR4‐high ovarian cancer, while AMD3100 combined with cisplatin demonstrates improved antitumor activity in CXCR4‐high tumors. These results suggest that further clinical translation of this CXCR4‐targeted theranostic approach could be highly valuable for improving ovarian cancer management.

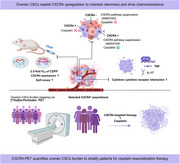

## 1 Introduction

Cytoreductive surgery combined with platinum‐based chemotherapy remains the standard treatment for ovarian cancer. Over 70% of patients develop acquired chemoresistance after an initial response, resulting in a 5‐year survival rate of less than 50% [1, 2]. Tumor heterogeneity is a major contributor to this therapeutic failure, particularly the dynamic evolution of cancer stem cells (CSCs) under chemotherapy‐induced selective pressure [3, 4, 5]. Ovarian CSCs can evade cytotoxic therapy through multiple mechanisms, including enhanced self‐renewal signaling [6], efficient DNA damage repair [7], metabolic adaptability [8], and cell cycle quiescence [9]. Moreover, conventional chemotherapy preferentially targets rapidly proliferating cancer cells, which may inadvertently enrich ovarian CSC populations [10]. Therefore, precisely targeted therapeutics that effectively eradicate ovarian CSCs are desirable to improve the treatment outcomes.

Therapeutic strategies targeting tumor‐initiating CSCs have primarily focused on exploiting cell‐surface markers to specifically identify and distinguish these cells. C‐X‐C chemokine receptor type 4 (CXCR4) is a seven‐transmembrane G‐protein‐coupled receptor that binds its cognate ligand CXCL12 and participates in diverse physiological processes, including embryonic development [11], homeostasis of hematopoietic stem cells [12], and the innate immune system [13]. As reported, CXCR4 was abnormally upregulated in multiple solid tumors and predicted patients’ prognosis [14]. CXCR4 could promote tumor progression, angiogenesis, and metastasis in a wide range of solid malignancies [15, 16, 17]. Importantly, CXCR4 has been widely identified as a functional CSC marker in various cancer types [18, 19], including gastric, prostate, pancreatic, renal, and lung cancers [20, 21, 22, 23, 24]. In ovarian cancer, elevated CXCR4 expression has been associated with tumor proliferation, metastasis, and mesenchymal characteristics [25, 26]. With the development of a series of CXCR4‐targeted agents in clinical trials, AMD3100 was the first CXCR4 antagonist approved by the Food and Drug Administration (FDA) [27]. It could impede tumor progression and enhance chemotherapy sensitivity of ovarian cancer [28, 29]. These findings suggest that CXCR4 may represent a prominent therapeutic target for overcoming chemoresistance. However, the practical clinical application of CXCR4‐directed therapy requires reliable methods to identify CXCR4‐high tumors and to stratify patients most likely to benefit.

Recent advances in molecular imaging have provided new opportunities for noninvasive characterization of tumor biology. Positron emission tomography/computed tomography (PET/CT), based on the specificity of molecular receptors, enables sensitive in vivo visualization of molecular targets [30, 31]. CXCR4‐directed PET imaging has been developed by conjugating radioisotopes to CXCR4 inhibitors and has been employed as a diagnostic tool in multiple solid tumors [32]. The PET agent [^68^Ga]Ga‐Pentixafor, which served as a high‐affinity ligand of CXCR4 [33], has allowed noninvasive assessment of CXCR4 expression burden in many hematologic and solid malignancies, including multiple myeloma, leukemia, lung cancer, and glioblastoma [34, 35, 36, 37]. Recent literature has reported that identification of CXCR4 with [^68^Ga]Ga‐Pentixafor plays an essential role in subtyping primary aldosteronism and in quantifying the target in vivo before therapy initiation [38]. CXCR4‐targeted PET/CT could aid in visualizing CXCR4 expression and its niche, including chemotherapy‐resistant tumors, to further guide effective treatment interventions. However, the application of [^68^Ga]Ga‐Pentixafor PET/CT in targeting CSCs and guiding clinical treatment of ovarian cancer is yet an unexplored approach.

This investigation supported the notion that ovarian CSCs exploit CXCR4 upregulation to maintain stemness and drive chemoresistance, and proposed a CXCR4‐based theranostic paradigm. Our findings provided functional evidence that CXCR4 defines a self‐renewing CSC population through limiting‐dilution and sphere‐formation assays. Transcriptomic profiling of SK‐3rd versus control SK‐NS cells revealed a CXC‐centered activation of NF‐κB/IL‐17/TNF inflammatory circuitry that sustains stemness. Leveraging this mechanistic insight, we engineered a CXCR4‐PET ([^68^Ga]Ga‐Pentixafor) for noninvasive quantification of ovarian CSCs burden. We also demonstrated the feasibility of PET‐guided combination therapy with the CXCR4 inhibitor AMD3100 and cisplatin, revealing differential therapeutic responses between CXCR4‐high and CXCR4‐low PDX models. Together, these advances go beyond prior studies that have described CXCR4 expression or signaling and support a clinically translatable “CXCR4 theranostic” paradigm for ovarian cancer. It could help visualize and manage residual resistant stem cell reservoirs in vivo, possibly steering ovarian cancer treatment toward more targeted stem cell eradication and away from purely empirical chemotherapy.

## 2 Results

### 2.1 Elevated CXCR4 Expression is Associated with Ovarian Cancer Progression and Chemoresistance

**FIGURE 1:**
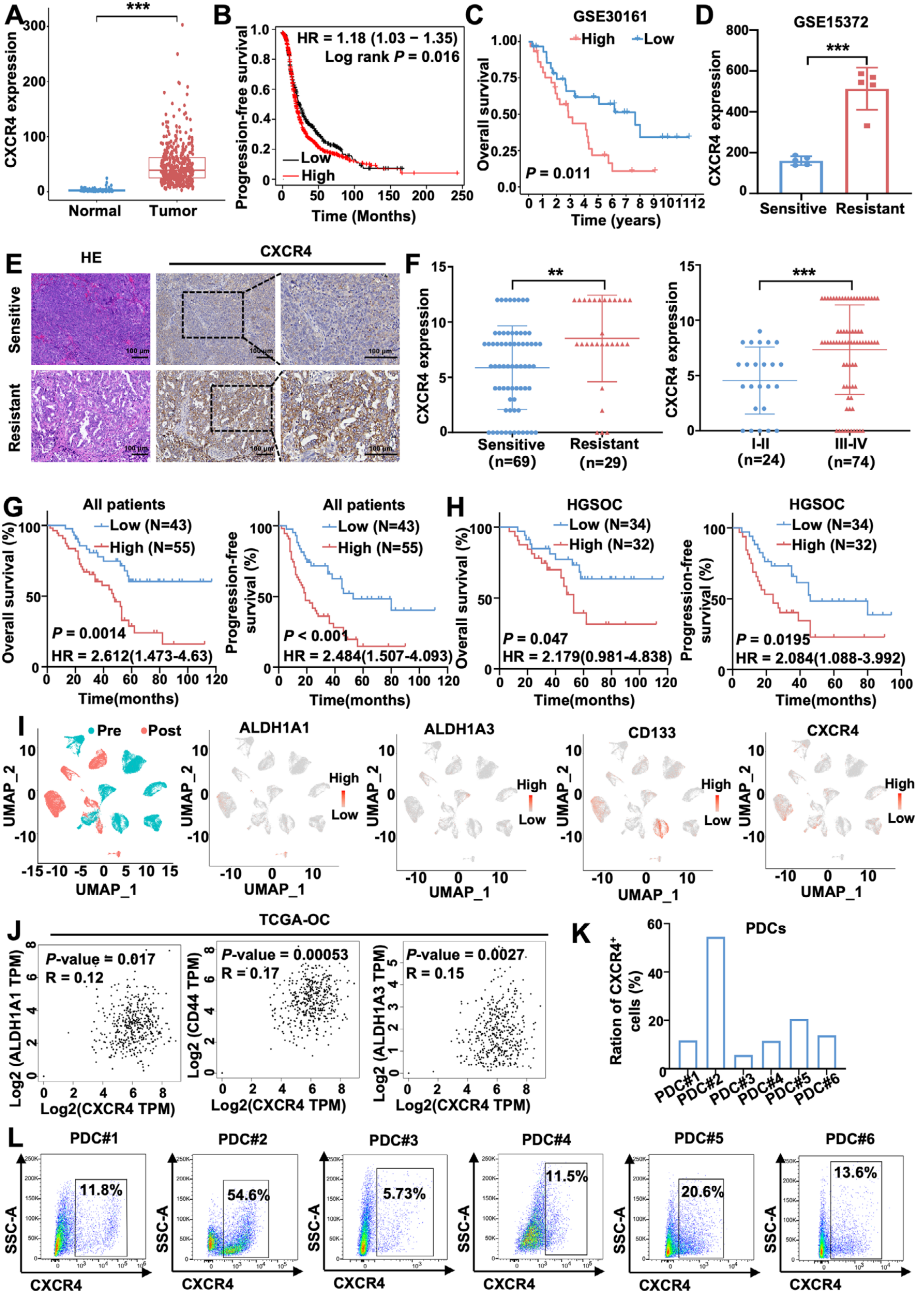
CXCR4 expression is elevated in chemoresistant ovarian cancer tissues and correlates with adverse clinical features. (A) mRNA expression level of CXCR4 in patients with ovarian cancer from TCGA (*n* = 401) and normal tissues from GTEx (*n* = 88). (B) Progression‐free survival (PFS) analysis of ovarian cancer patients with high and low CXCR4 expression in the Kaplan–Meier plotter database. (C) overall survival (OS) analysis of ovarian cancer patients with high and low CXCR4 expression in the GSE30161 database. (D) mRNA expression level of CXCR4 in samples from the GSE15372 database. (E) Representative IHC staining of CXCR4 expression in ovarian cancer tissues. Scale bar = 100 µm. (F) Expression level of CXCR4 in different chemotherapy response groups and FIGO stages. OS and PFS of all (G) & HGSOC (H) ovarian cancer patients with high and low CXCR4 expression. (I) Uniform manifold approximation and projection (UMAP) analysis for single‐cell sequencing of eight pre‐treatment and six post‐treatment malignant ascites or pleural samples from the GSE158722 dataset. (J) Relationship between CXCR4 and CSC markers in the TCGA‐ovarian cancer cohort with GEPIA analysis. (K, L) Different proportions of CXCR4^+^ cells in six patient‐derived cells (PDCs): PDC#1‐PDC#6. Data are presented as mean ± SD. ^*^ *p* < 0.05, ^**^ *p* < 0.01, ^***^ *p* < 0.001.

Due to the overexpression of CXCR4 in ovarian cancer tissues compared with normal tissues (*p* < 0.05) (Figure 1A), the relationship between CXCR4 and chemoresistance was subsequently explored with clinical samples. We detected CXCR4 expression in ovarian cancer using the Cancer Genome Atlas (TCGA) and Genotype‐Tissue Expression (GTEx) databases. It suggested that an elevated level of CXCR4 was correlated with worse progression‐free survival (PFS) in ovarian cancer patients with Kaplan–Meier (KM) Plotter (HR = 1.18, *p* = 0.016) (Figure 1B) and overall survival (OS) of ovarian cancer patients from GSE30161 (*p* = 0.011) (Figure 1C). Further data mining showed that CXCR4 was upregulated in the CDDP‐resistant group compared with the CDDP‐sensitive group in GSE15372 datasets (*p* < 0.05) (Figure 1D). In agreement with the above results, immunohistochemistry (IHC) staining revealed that high CXCR4 expression was associated with chemotherapy resistance (*p* < 0.001), FIGO stages (III‐IV) (*p* < 0.001) and recurrence (*p* = 0.002) (Table S1), and CXCR4 expression was significantly increased in patients with chemotherapy resistance and FIGO III‐IV stages (all *p* < 0.05) (Figure 1E,F). Moreover, Kaplan‐Meier survival analysis indicated that high CXCR4 expression was associated with worse PFS as well as OS in all ovarian cancer patients and HGSOC (all *p* < 0.05) (Figure 1G,H). Based on these findings, high CXCR4 expression is associated with chemoresistance, advanced FIGO stage, disease recurrence, and shorter progression‐free and overall survival in ovarian cancer.

Besides, we analyzed the single‐cell RNA sequencing (scRNA‐seq) dataset GSE158722, which contained ovarian cancer patients receiving platinum‐based chemotherapy. The findings suggested that stem cell markers (ALDH1A1, ALDH1A3, CD133) and CXCR4 were increased in post‐treatment malignant ascites or pleural samples (Figure 1I), suggesting that chemotherapy enhanced CSCs enrichment and CXCR4 expression. Moreover, CXCR4 expression displayed a positive correlation with CSC markers ALDH1A1 (*R* = 0.12, *p* = 0.017), CD44 (*R* = 0.17, *p* = 0.00053), and ALDH1A3 (*R* = 0.15, *p* = 0.0027) in the GEPIA database, and CXCR4^+^ cells show a concomitant increase with ovarian CSC markers following chemotherapy (Figure 1J). We explored CXCR4 expression in six patient‐derived cells (PDCs) and found that the proportions of CXCR4^+^ cells varied among the PDCs (Figure 1K,L). Together, these findings suggest that CXCR4 may help sustain ovarian CSC properties and mediate chemoresistance.

### 2.2 Upregulation of CXCR4 in Ovarian CSCs

**FIGURE 2:**
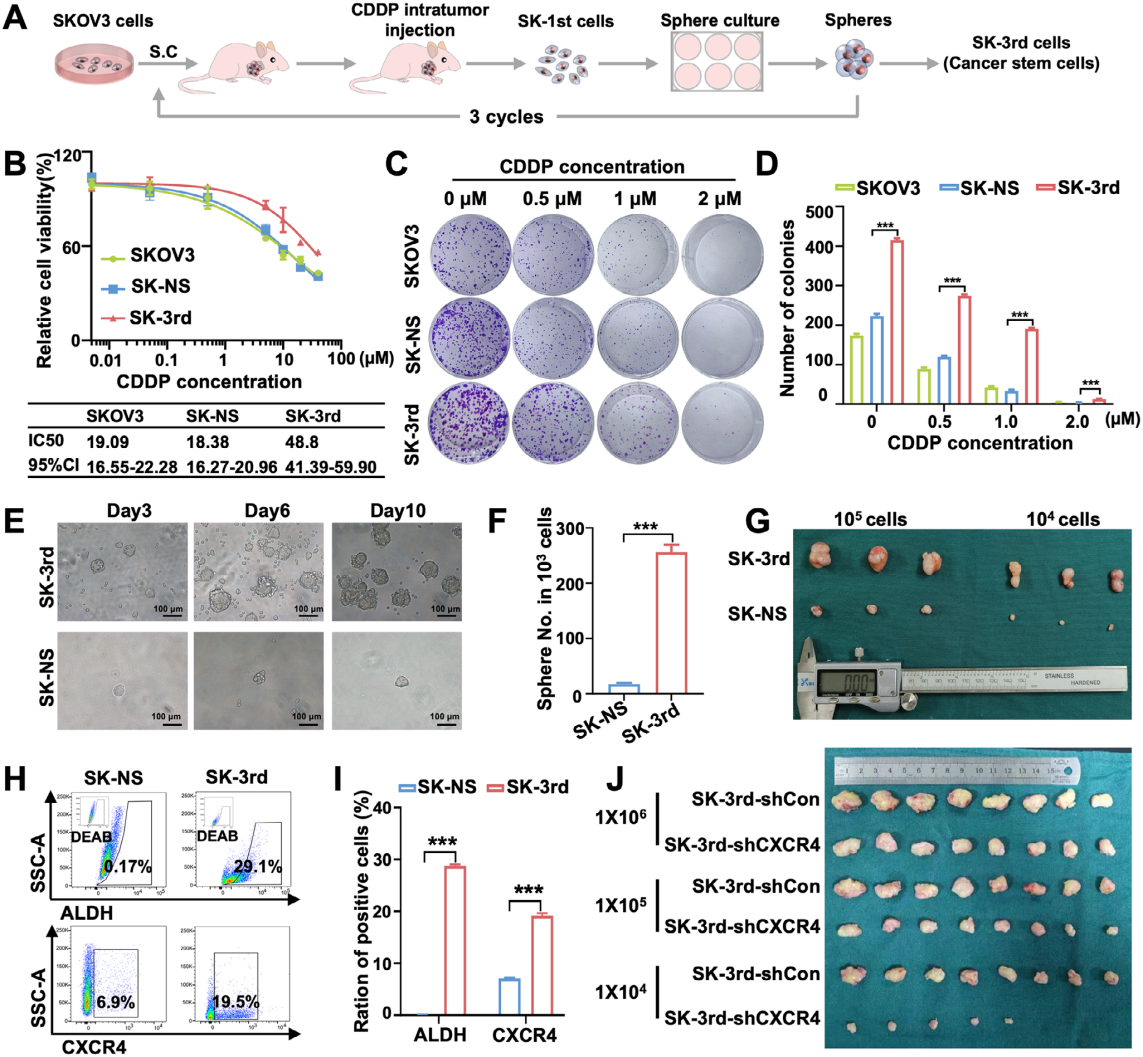
Enrichment of CXCR4‐positive cells in cisplatin‐resistant and sphere‐forming ovarian cancer models. (A) Schematic illustration of the establishment of ovarian cancer stem cell SK‐3rd. (B) Cell viability of SK‐3rd cells and control groups treated with CDDP was detected by MTT assay (*n* = 6). (C) Colony formation of SK‐3rd cells and control groups with different treatments was determined by colony formation assay (*n* = 3). (D) Statistical histogram of colony formation assay in SK‐3rd and the control groups (*n* = 3). (E) Representative images and (F) statistical histogram of spheres formed by 10^3^ SK‐3rd cells and 10^3^ SK‐NS cells (*n* = 3). Scale bar = 100 µm. (G) Representative images of solid tumors isolated from mice (*n* = 3 per group). (H, I) Percentage of ALDH^+^ and CXCR4^+^ subpopulations in SK‐NS and SK‐3rd cells detected by flow cytometry (*n* = 3). (J) The tumors harvested at the end of the limited dilution tumorigenicity assay (*n* = 8 per group). SK‐NS represents tumor cells obtained from xenografts treated with 0.9% sterile saline; SK‐3rd represents tumor cells obtained from third‐generation xenografts treated with CDDP; SK‐3rd‐shCXCR4 represents a stable cell line derived from SK‐3rd cells with CXCR4 knockdown; and SK‐3rd‐shCon serves as the control group without any gene knockdown. Data are presented as mean ± standard deviation (SD). ^**^ *p* < 0.01, ^***^ *p* < 0.001.

Given that CXCR4 expression is concomitantly elevated with the enrichment of CSC markers in chemoresistant ovarian cancer patients, we hypothesize that ovarian CSCs acquire cisplatin resistance through CXCR4 upregulation. To further investigate the functional relationship between CXCR4 and the ovarian CSC phenotype, we established a model of cisplatin treatment in SKOV3 cells. Female BALB/c‐nude mice subcutaneously implanted with SKOV3 cells were treated with CDDP intratumorally, combined with in vitro interval suspension culture, for three cycles of selection; the control group received saline. Cells from the third passage isolated from the third‐generation mice were identified as SK‐3rd (Figure 2A). We first confirmed the drug resistance of SK‐3rd cells. Cell viability assay showed that IC_50_ of CDDP in the SK‐3rd cells increased approximately 2.5‐fold compared with that in control SK‐NS cells and the parental SKOV3 cells (Figure 2B). Colony formation assay demonstrated that clone numbers formed by SK‐3rd cells were much higher than those of control SK‐NS cells and parental SKOV3 cells at higher CDDP concentrations, confirming increased resistance to CDDP in SK‐3rd cells (Figure 2C,D).

Next, sphere‐formation assays showed that SK‐3rd cells (10^3^ per well) generated significantly more tumor spheres than SK‐NS cells (*p* < 0.05), confirming their enhanced self‐renewal capacity and sustained stemness (Figure 2E,F). Subsequently, we examined the tumor‐initiating capacity of SK‐3rd cells with xenograft transplantation assays. The results revealed that SK‐3rd cells exhibited enhanced tumor initiation capacity in BALB/c‐nude mice compared with that in control SK‐NS cells (Figure 2G), manifested by much larger tumor volume and weight in mice bearing SK‐3rd cells (all *p* < 0.05) (Figure S1). Flow cytometric analysis showed that the percentage of ALDH^+^ subpopulation and CXCR4^+^ subpopulation in SK‐3rd cells was significantly increased compared to those in SK‐NS cells, respectively (all *p* < 0.05) (Figure 2H,I).

The gold standard for CSC testing is the limited dilution and tumorigenesis assay in vivo. As illustrated in Figure 2J, when the inoculated cell number was reduced to 1 × 10^4^, the control group retained a 100% tumor‐initiation rate. In contrast, the CXCR4‐knockdown group exhibited a marked decline to 62.50% (5/8), accompanied by significant reductions in residual tumor volume and weight. The results indicated that CXCR4 does not merely correlate with tumor bulk but directly attenuates the intrinsic tumor‐initiating capacity of SK‐3rd, functionally validating the proposition that SK‐3rd acquires tumorigenic and chemoresistant advantages through CXCR4 upregulation. Here, we validated the stemness of SK‐3rd cells through their pronounced self‐renewal capacity and robust tumorigenicity, confirming that cisplatin treatment effectively enriches for CSCs. Accordingly, SK‐3rd cells serve as a bona fide model of ovarian cancer stem‐like cells whose stemness is sustained by CXCR4 upregulation.

Besides, we also found an increased proportion of CXCR4^+^ subpopulation in spheres derived from ES‐2 cells, which exhibited a strong CSCs phenotype, compared with the counterpart adherent cells (all *p* < 0.05) (Figure S2A,B). Then, CXCR4^+^ cells and CXCR4^−^ cells were sorted from spheres formed by ES‐2 cells with fluorescence‐activated cell sorting (FACS) (Figure S2C). Sphere formation assays revealed an increased number of spheres formed by CXCR4^+^ ES‐2 cells compared with CXCR4^−^ ES‐2 cells (all *p* < 0.05) (Figure S2D), suggesting that CXCR4^+^ cells presented enhanced self‐renewal capacity. We further observed that CXCR4^+^ cells possessed enhanced tumor initiation capacity in BALB/c‐nude mice; in contrast, CXCR4^−^ cells could barely form tumors (all *p* < 0.05) (Figure S2E). Collectively, these results suggest that upregulation of CXCR4 plays a vital role in maintaining stemness in ovarian CSCs, including self‐renewal capacity, tumor initiation, and progression.

### 2.3 RNA‐seq Analysis of SK‐NS and SK‐3rd Cells

**FIGURE 3:**
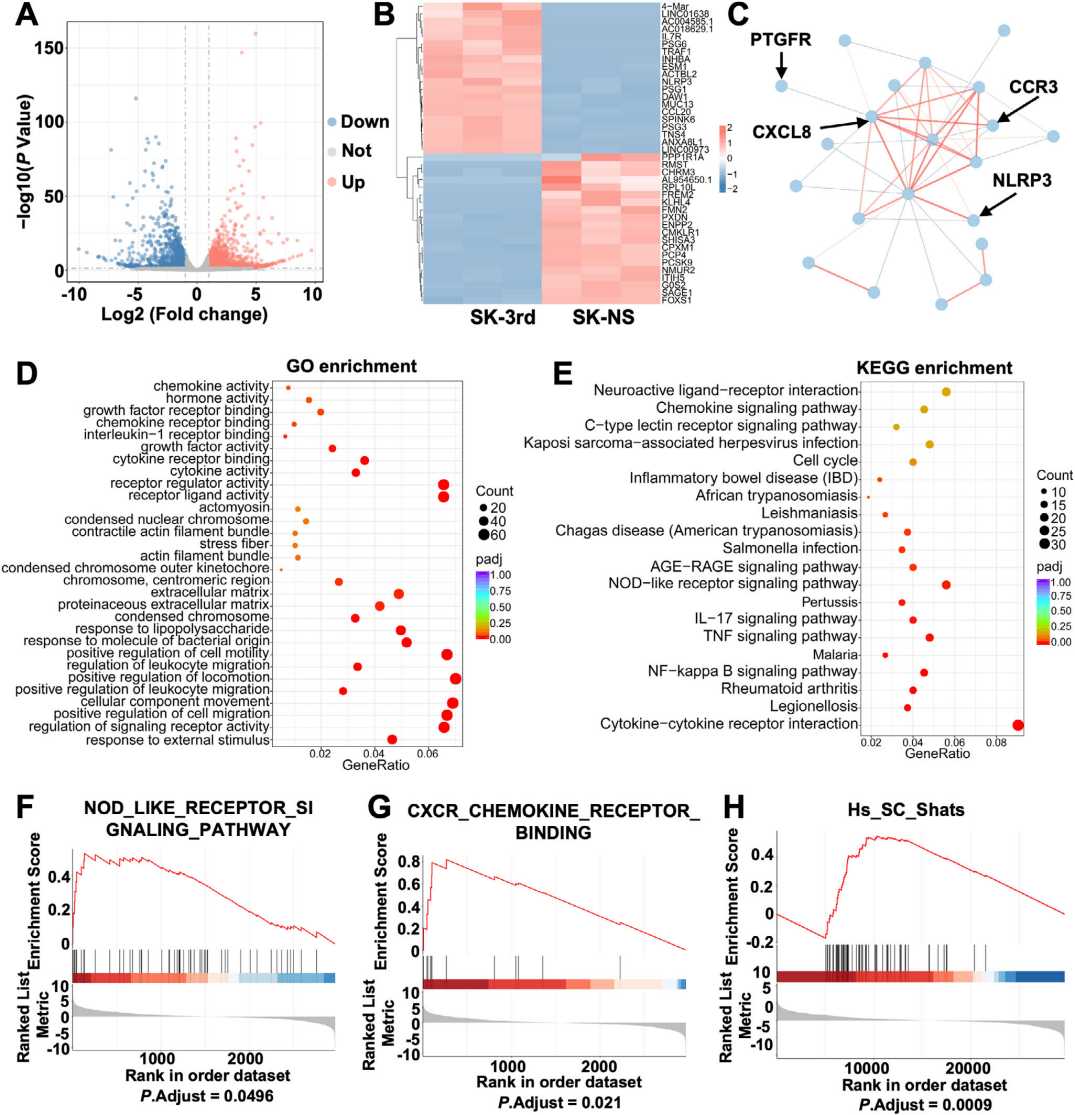
Transcriptomic profiling of SK‐NS and SK‐3rd cells by RNA sequencing. (A) DEGs shown by volcano plots. Blue dots represented downregulated DEGs, red dots represented upregulated DEGs, and gray dots represented genes with no statistical significance. (B) Differential expression profiles for SK‐NS and SK‐3rd cells with a heatmap (*n* = 3). Rows indicated individual samples, and columns indicated differentially expressed genes. (C) Protein–protein interaction networks of DEGs. (D) Gene ontology (GO) enrichment analysis of DEGs between SK‐NS and SK‐3rd cells. (E) Kyoto Encyclopedia of Genes and Genomes (KEGG) enrichment analysis of DEGs between SK‐NS and SK‐3rd cells. (F) Gene Set Enrichment Analysis (GSEA) analysis revealed that the NOD‐like receptor signaling pathway gene set was enriched in SK‐3rd cells. (G) GSEA analysis revealed that the gene set of CXCR chemokine receptor binding was enriched in SK‐3rd cells. (H) GSEA analysis showed that the stemness gene set of Hs_SC_Shats was enriched in SK‐3rd cells.

To determine the molecular mechanism underlying SK‐3rd stemness, high‐throughput RNA sequencing (RNA‐Seq) was performed on SK‐NS and SK‐3rd cells. Transcriptomic profiling revealed a pronounced reprogramming landscape within SK‐3rd cells. Volcano plot analyses identify 2821 differentially expressed genes (1440 upregulated, 1381 downregulated) that cleanly segregate SK‐3rd from SK‐NS controls (Figure 3A). The upregulated cohort is enriched for inflammatory mediators and membrane‐associated factors (CCL20, MUC13, NLRP3, ANXA8L1), whereas differentiation‐associated transcripts (ENPP2, PCP4, KLHL4, FOXS1, PPP1R1A) are selectively silenced (Figure 3B). Furthermore, protein–protein interaction mapping positions CXCL‐family chemokines and innate immune sensors at the core of this network, underscoring a cytokine‐centric circuitry (Figure 3C). Gene ontology (GO) enrichment analysis shows that the DEGs are significantly concentrated in functions such as modulation of signaling receptor activity, cell motility, receptor‐ligand activity, and cytokine/IL‐1 receptor binding, suggesting that SK‐3rd cells sustain stemness by intensifying cell‐microenvironment communication (Figure 3D). Kyoto Encyclopedia of Genes and Genomes (KEGG) enrichment analysis further narrows the focus to inflammatory pathways, including cytokine–cytokine receptor interaction, NF‐κB, IL‐17, TNF, and NOD‐like receptor, all of which have been extensively implicated in CSC self‐renewal and chemoresistance (Figure 3E,F). In addition, Gene Set Enrichment Analysis (GSEA) further reveals coordinate enrichment of CXCR chemokine receptor binding, and the stem gene set (Hs_SC_Shats) acquired from the StemChecker database was also observed and enriched in SK‐3rd cells (Figure 3G,H). Overall, the RNA‐seq data suggest that, at the transcriptomic level, SK‐3rd ovarian CSCs may concurrently bolster stem‐like properties and cisplatin resistance through a CXC‐centered chemokine‐inflammation pathways that cooperates with NF‐κB/IL‐17/TNF and other signaling cascades. Although NLRP3 and ANXA8L1 were among the top DEGs, subsequent PPI mapping and GSEA analyses positioned CXCR4 and the CXCL family of chemokines at the core of the inflammatory circuitry sustaining stemness. Thus, CXCR4 was prioritized for its dual role as both a CSC marker and a targetable therapeutic node.

### 2.4 Effects of CXCR4 Inhibition on Stemness and CDDP Sensitivity

**FIGURE 4:**
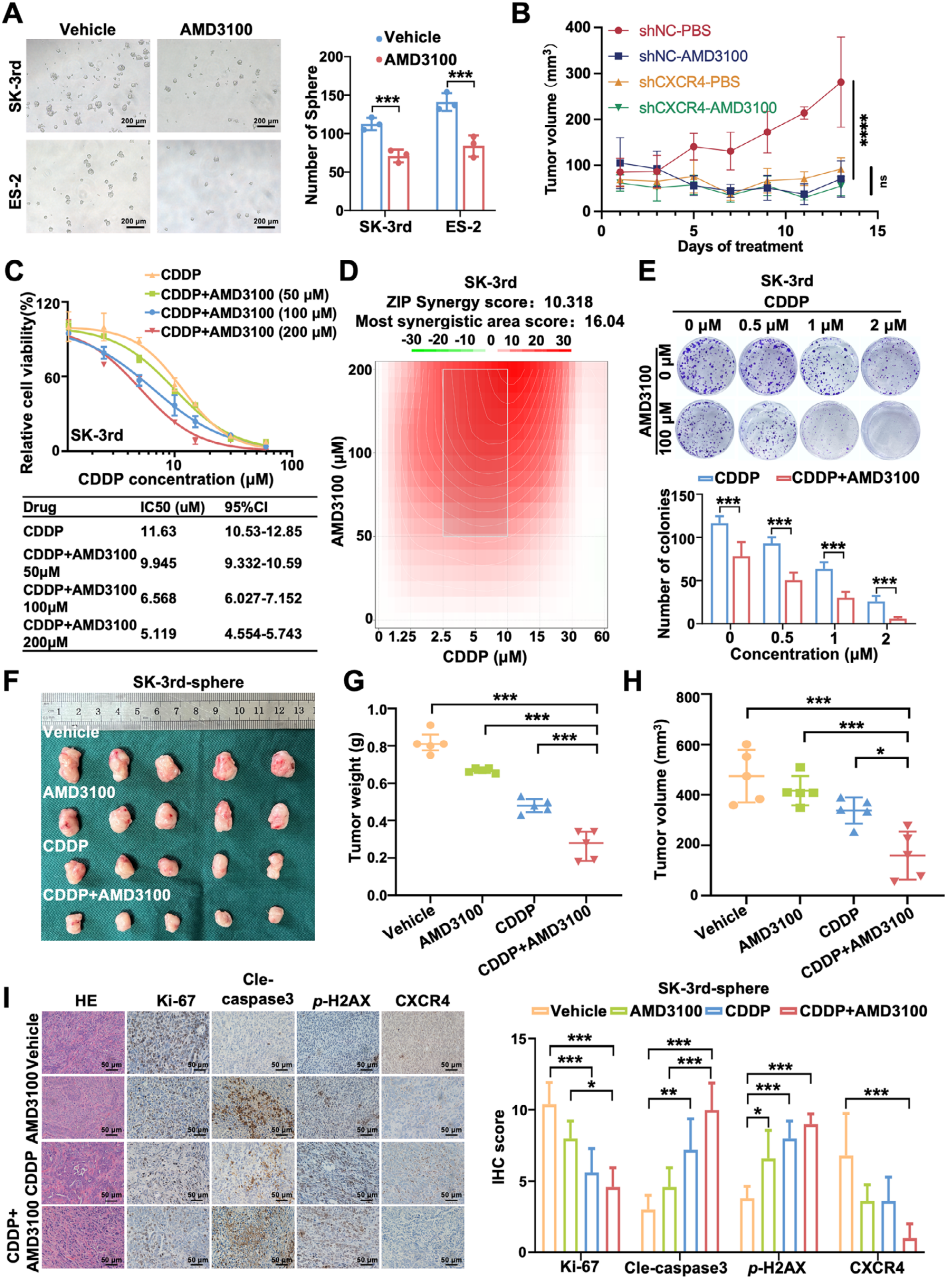
CXCR4 inhibition reduces sphere formation and enhances cisplatin sensitivity in ovarian cancer cells. (A) Sphere formation ability of SK‐3rd and ES‐2 cells treated with vehicle and AMD3100 (100 µM), respectively (*n* = 3). Scale bar = 200 µm. (B) Tumor volume of AMD3100 treatments assay, tumor volume = 0.5 × length ×width^2^ (*n* = 6). (C) Cell viability of SK‐3rd cells treated with AMD3100 and CDDP determined by MTT assay (*n* = 3). (D) Synergistic cytotoxic effects of AMD3100 and CDDP on SK‐3rd cells analyzed with SynergyFinder2.0. (E) Colony formation assay of SK‐3rd cells treated with AMD3100 (0 µM and100 µM) and CDDP (0, 0.5, 1, and 2 µM) (*n* = 3). (F) Represent images of tumors derived from mice bearing SK‐3rd cells treated with vehicle, CDDP, AMD3100, and CDDP + AMD3100, respectively (*n* = 5 per group). Tumor weight (G) and tumor volume (H) in mice bearing SK‐3rd cells treated with vehicle, CDDP, AMD3100, and CDDP + AMD3100 (*n* = 5 per group). (I) H&E staining of tumors and immunohistochemistry for Ki‐67, cle‐caspase3, *p*‐H2AX, and CXCR4 in xenografts from mice treated with vehicle, CDDP, AMD3100, and CDDP + AMD3100. Scale bar = 50 µm. Data are presented as mean ± SD. ^*^ *p* < 0.05, ^**^ *p* < 0.01, ^***^ *p* < 0.001.

To test the hypothesis that CXCR4 upregulation enables ovarian CSCs to acquire and sustain chemoresistance, we next investigated the effects of CXCR4 inhibition on ovarian CSC characteristics. We evaluated the therapeutic benefit of combining a CXCR4 antagonist with CDDP. AMD3100 (Plerixafor) was the first small molecule CXCR4 inhibitor approved by the Food and Drug Administration (FDA) and is clinically feasible. Consistent with our earlier findings, the sphere‐formation assay showed that AMD3100 elicited a significantly greater reduction in sphere formation in SK‐3rd (37.98%) and ES‐2 (40.43%) cells compared with their respective controls (all *p* < 0.05), underscoring impairment of stemness (Figure 4A). Flow cytometry suggested that percentages of CXCR4^+^ population were also decreased in SK‐3rd and ES‐2 cells treated with AMD3100 (all *p* < 0.05) (Figure S3). Therapeutic intervention revealed that AMD3100 markedly attenuates both tumor mass and volume compared with PBS‐treated controls, an effect that is completely abrogated upon CXCR4 knockdown, where AMD3100 performs no better than vehicle. Notably, CXCR4 depletion alone achieved a therapeutic effect comparable to that of AMD3100 monotherapy, establishing that AMD3100's antitumor activity is strictly CXCR4‐dependent (Figure 4B, Figure S4).

The effect of CXCR4 inhibition with AMD3100 on ovarian CSCs' chemoresistance was subsequently examined in combination with CDDP treatment. Cell viability assay showed that AMD3100 could boost CDDP sensitivity of CDDP‐resistant SK‐3rd cells with a decrease in IC_50_ values compared to that in CDDP treatment alone, confirming the sensitizing effect of AMD3100 on CDDP (*p* < 0.05) (Figure 4C). Similar results were also observed in ES‐2 cells (*p* < 0.05) (Figure S5A). SynergyFinder2.0 was further utilized to analyze the combinatorial effects of the two drugs. The comprehensive synergy scores and maps of the drug combination were shown in Figure 4D and Figure S5B. The comprehensive synergy scores for AMD3100 and CDDP (10.318 in SK‐3rd and 10.526 in ES‐2 cells) indicated that the combination exhibited synergistic cytotoxicity across the tested concentration range. We further analyzed the effects of AMD3100 and CDDP on the clone formation capacity of SK‐3rd and ES‐2 cells. We found that AMD3100 significantly reduced the number of colonies formed by SK‐3rd cells and ES‐2 cells treated with CDDP, suggesting that AMD3100 rendered ovarian cancer sensitive to CDDP‐induced growth inhibition (Figure 4E and Figure S5C). The antitumor effect of CXCR4 inhibitor and CDDP were further investigated in vivo, the results demonstrated that average tumor volume in AMD3100 + CDDP group was 159.36 ± 94.87 mm^3^ with tumor suppression rate (TSR) of 66.38%, which was much higher than that in CDDP group (337.63 ± 51.98 mm^3^) with TSR of 28.77% and AMD3100 group (416.77 ± 58.31 mm^3^) with TSR of 12.08%, respectively (Figure 4F,G; Table S3). The calculated Q value was 1.78 for the AMD3100 + CDDP group, indicating a synergistic therapeutic effect of AMDA3100 in combination with CDDP for ovarian cancer treatment (Table S3). Average tumor weight in the AMD3100 + CDDP group (0.27 ± 0.08 g) was significantly decreased compared with that in the CDDP group (0.48 ± 0.04 g) and AMD3100 group (0.67 ± 0.01 g), indicating that AMD3100 enhanced CDDP sensitization of ovarian cancer cells (Figure 4H). H&E staining showed nuclear shrinkage and disappearance in the AMD3100 + CDDP group. IHC staining also demonstrated that the level of CXCR4, Ki‐67, *p*‐H2AX was inhibited, and cle‐caspase3 was enhanced with AMD3100 and CDDP combination (Figure 4I), and similar results were obtained with BALB/c‐nude mice bearing ES‐2 sphere (Figure S5D–H; Table S4).

Collectively, genetic or pharmacologic ablation of CXCR4 markedly attenuated the self‐renewal and in vivo tumorigenicity of ovarian CSCs and concurrently restored CDDP sensitivity. These data supported CXCR4 upregulation as an essential molecular mechanism of ovarian CSCs‐driven chemoresistance, providing a mechanistic rationale for integrating CXCR4 antagonists with platinum‐based therapy in ovarian cancer.

### 2.5 CXCR4‐PET Imaging of Chemoresistant Ovarian Cancer

To prospectively chart the emergence of chemoresistance and enable timely, targeted intervention, we aimed to develop a positron emission tomography/computed tomography (PET/CT) imaging method to quantify the burden of ovarian CSCs noninvasively. ^18^F‐FDG PET/CT remains the clinical mainstay and gold standard for the nuclear imaging of ovarian cancer, however its specificity is compromised by a substantial false‐positive rate (19%). The fibroblast activation protein (FAP) targeted radiotracer [^68^Ga]Ga‐FAPI‐04 has emerged as a promising tumor‐agnostic alternative that effectively addresses this limitation. Moreover, emerging evidence implicates FAP in the chemoresistance acquisition in ovarian cancer. [^68^Ga]Ga‐Pentixafor is another radiotracer predominantly used in the clinical imaging assessment of lymphoma and multiple myeloma, owing to its exceptional CXCR4‐targeting properties.

**FIGURE 5:**
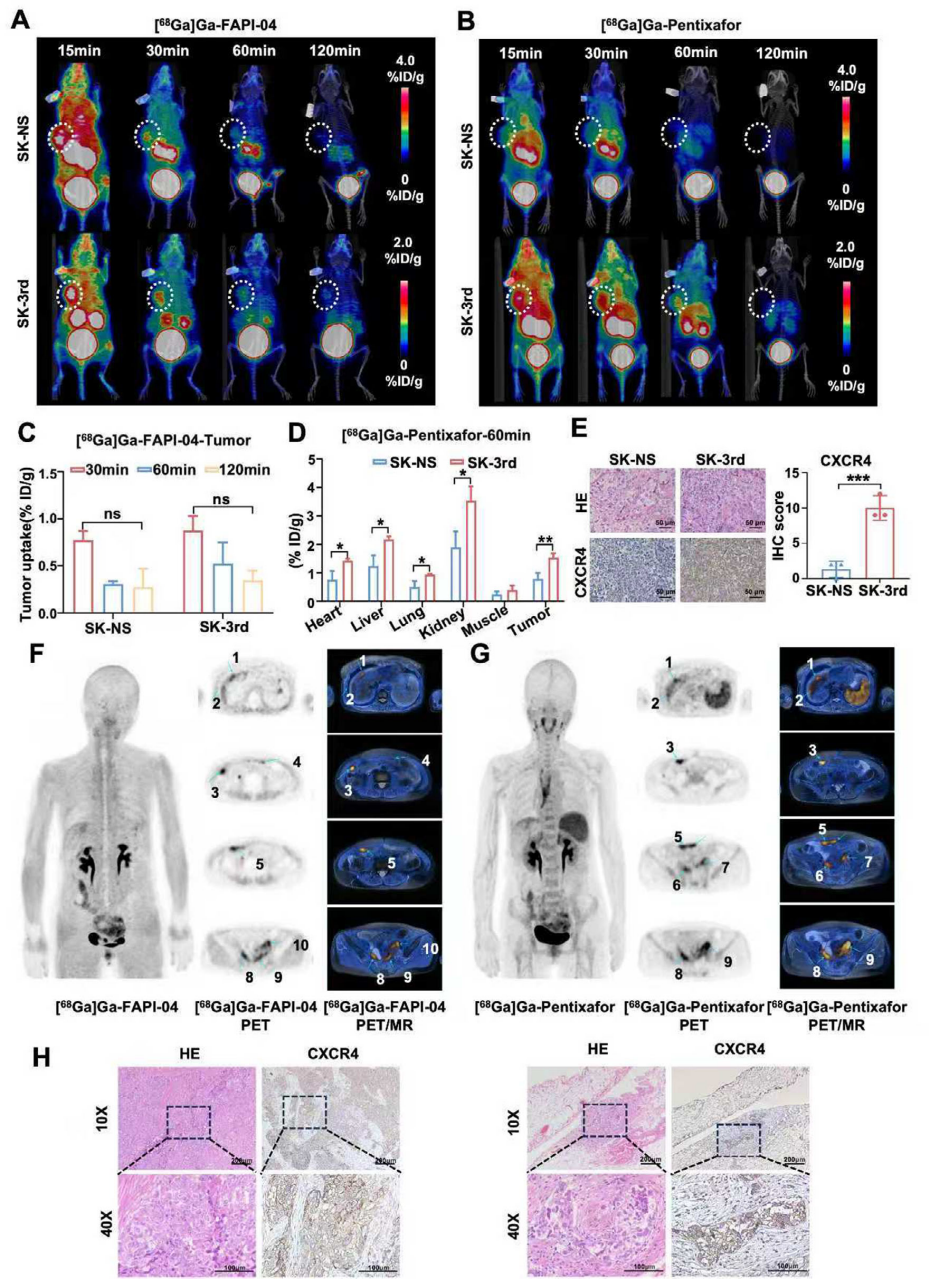
CXCR4‐PET specifically recognizes SK‐3rd and ovarian cancer lesions. Representative [^68^Ga]Ga‐FAPI‐04 PET/CT images (A) and [^68^Ga]Ga‐Pentixafor PET/CT images (B) for SK‐NS and SK‐3rd xenograft models (*n* = 3 per group). (C) Biodistribution of [^68^Ga]Ga‐FAPI‐04 PET/CT in SK‐NS and SK‐3rd models at 30 min, 60 min, and 120 min (*n* = 3 per group). Ns: no significance. (D) Biodistribution of [^68^Ga]Ga‐Pentixafor in SK‐NS and SK‐3rd models at 60 min (*n* = 3 per group). (E) Representative images of HE staining and IHC staining of CXCR4 expression in the indicated groups (*n* = 3 per group), Scale bar = 50 µm. Images of [^68^Ga]Ga‐FAPI‐04 (F) and [^68^Ga]Ga‐Pentixafor (G) in patient with ovarian cancer, left: maximum‐intensity‐projection (MIP) of PET image, middle: transaxial PET images, right: corresponding transaxial PET/ MR fusion images. Major tumor sites of uptake are labeled: hepatic capsule (1,2), peritoneum (3,4), omentum and mesentery (5), pelvic peritoneal recession (6,7), adnexal masses (8,9,10). (H) Representative images of HE staining and CXCR4 expression in different omental ovarian cancer tissues with immunohistochemistry. Data are presented as mean ± SD. ^*^ *p* < 0.05, ^**^ *p* < 0.01, ^***^ *p* < 0.001.

We established the subcutaneous xenografts bearing sphere formed by SK‐NS and SK‐3rd cells, representing low and high CXCR4 expression, respectively. Figure 5A,B shows the representative [^68^Ga]Ga‐FAPI‐04 and [^68^Ga]Ga‐Pentixafor PET/CT images of SK‐NS and SK‐3rd tumors. As shown in Figure 5C, there were no significant differences in tumor uptake of [^68^Ga]Ga‐FAPI‐04 between SK‐NS and SK‐3rd tumor models at all time points from 30 to 120 min (Figure S6A,B), T/M ratios of that as well (Figure S6C), indicating that [^68^Ga]Ga‐FAPI‐04 PET/CT imaging could not differentiate SK‐NS (low CXCR4 expression) and SK‐3rd (high CXCR4 expression) tumor models. Quantitative data from ROI analysis showed that the tumor uptake of [^68^Ga]Ga‐Pentixafor decreased over time (1.97 ± 0.61%ID/g at 30 min, 1.53 ± 0.15%ID/g at 60 min, 0.46 ± 0.38%ID/g at 120 min) in SK‐3rd tumor models, there was a rapid decrease (1.23 ± 0.06%ID/g at 30 min, 0.67 ± 0.17%ID/g at 60 min, 0.41 ± 0.31%ID/g at 120 min) in SK‐NS tumor models from 30 to 120 min (Figure S6D,E), indicating rapid blood clearance and short retention time in tumor. Remarkably, SK‐3rd tumors exhibited much higher and persistent radiotracer uptake of [^68^Ga]Ga‐Pentixafor with 1.53 ± 0.15%ID/g at 60 min compared with those in SK‐NS tumors with 0.67 ± 0.17%ID/g at 60 min (*p* < 0.05) (Figure 5D). Additionally, other organs, such as the heart, liver, lung, and kidney, displayed relatively higher accumulation and slower clearance of [^68^Ga]Ga‐Pentixafor in SK‐3rd tumor models compared with those in SK‐NS tumor models at 60 min (all *p* < 0.05) (Figure 5D). There were no significant differences in T/M ratios between SK‐NS and SK‐3rd tumor models (Figure S6F). Moreover, IHC staining confirmed higher CXCR4 expression in SK‐3rd xenograft tumors, which was significantly greater than in SK‐NS tumors (Figure 5E), indicating that probe signal intensity was positively correlated with CXCR4 expression. The results of the biodistribution study directly demonstrated differential uptake in xenograft tumors and organs across groups, indicating that [^68^Ga]Ga‐Pentixafor enabled the visualization of CXCR4‐high tumor burden and the noninvasive quantification of CXCR4 expression.

Moreover, a head‐to‐head PET/MR imaging with [^68^Ga]Ga‐FAPI‐04 and [^68^Ga]Ga‐Pentixafor in a patient with confirmed primary high‐grade serous ovarian cancer was performed. The safety of [^68^Ga]Ga‐Pentixafor was further demonstrated by the fact that no adverse symptoms were observed in the patient throughout the PET/MR imaging procedure and during the 2‐week follow‐up. The distribution of [^68^Ga]Ga‐FAPI‐04 and [^68^Ga]Ga‐Pentixafor in primary and distant tumor sites throughout the body is displayed with maximum intensity projections (MIP) in Figure 5F,G. The kidney and bladder showed the highest radioactivity, followed by the liver. The heart, brain, and muscle are also identified, with relatively lower activity. By reference to matched [^68^Ga]Ga‐FAPI‐04, the positive lesions, including primary tumors and metastatic tumors, could be clearly observed after injection of [^68^Ga]Ga‐Pentixafor intravenously. The uptake SUV_max_ of primary ovarian cancer lesions was 4.8–6.2 with [^68^Ga]Ga‐FAPI‐04 and 4.0–7.3 with [^68^Ga]Ga‐Pentixafor, respectively. The uptake SUV_max_ of metastatic ovarian cancer lesions (hepatic capsule, peritoneum, omentum, mesentery, and pelvic peritoneal recession) were 3.7–7.1 with [^68^Ga]Ga‐FAPI‐04 and 2.8–8.0 with [^68^Ga]Ga‐Pentixafor, respectively. Finally, CXCR4 expression was detected in omental lesions (Figure 5H). Prominent lesions with high uptake of [^68^Ga]Ga‐Pentixafor were CXCR4‐positive, suggesting concordant CXCR4 expression in tumor sites in vivo and ex vivo. Compared with FAPI‐04, the CXCR4‐targeted radiotracer [^68^Ga]Ga‐Pentixafor more effectively identifies tumors with high CXCR4 expression in preclinical ovarian cancer models. In patients, the probe achieves a comparable detection rate and thus holds promise for refining treatment selection to maximize therapeutic benefit.

### 2.6 [^68^Ga]Ga‐Pentixafor PET imaging in CXCR4‐High and CXCR4‐Low ovarian cancer models

**FIGURE 6:**
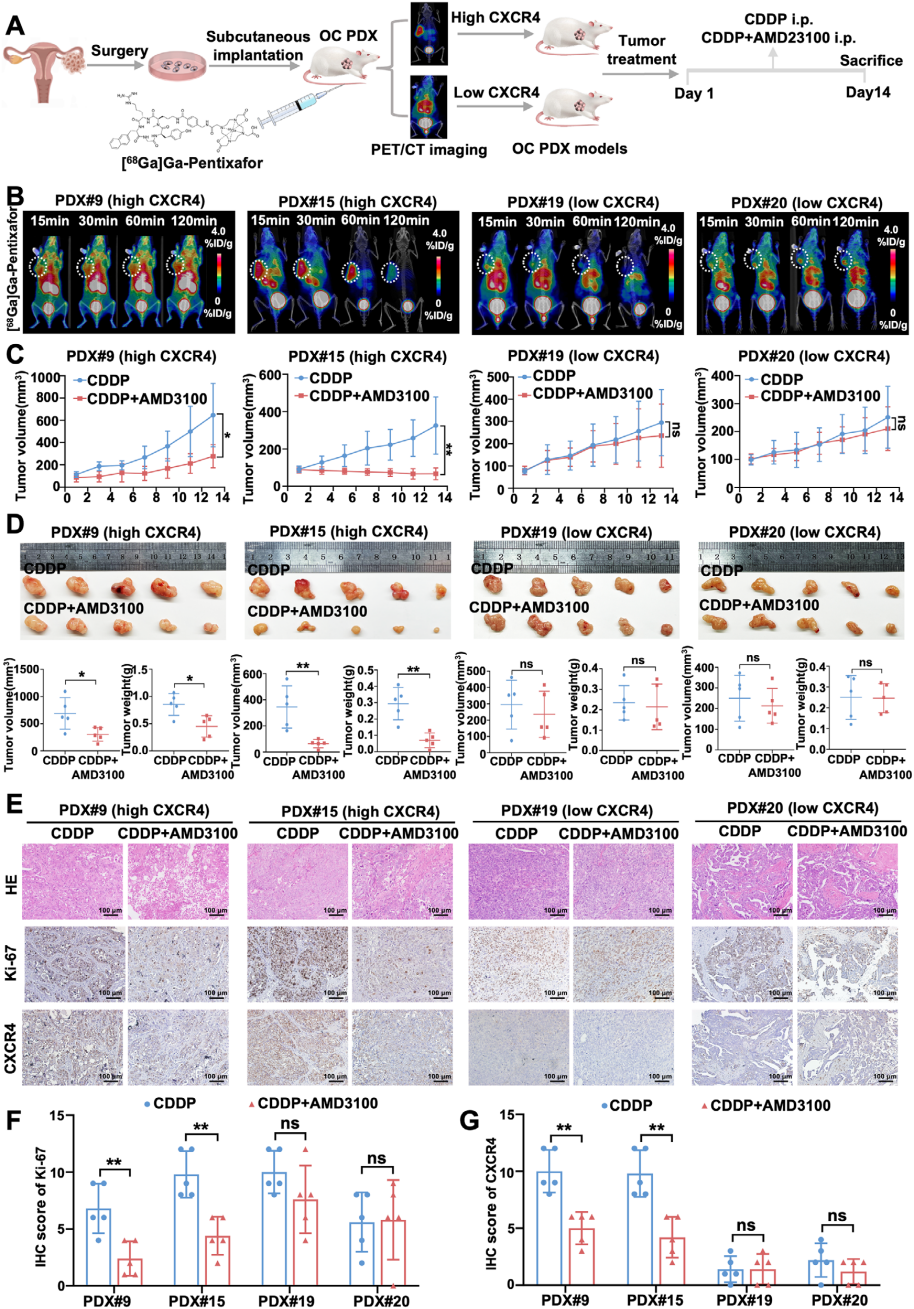
Effects of combined treatment of AMD3100 and CDDP on CXCR4‐low and ‐high PDX tumors. (A) Schematic illustration of the establishment of the ovarian cancer PDX models for in vivo imaging and therapy. (B) Representative images of accumulation of [^68^Ga]Ga‐Pentixafor in different organs of CXCR4‐high and ‐low PDX models. (C) Tumor growth curve in different experimental groups of mice (*n* = 5 per group). (D) Tumor volume and tumor weight of PDX tumor treated with CDDP and CDDP + AMD3100 (*n* = 5 per group). (E) Representative images of HE staining and expression of Ki‐67 and CXCR4 detected by immunohistochemical staining in the indicated ovarian cancer PDX tumors following the indicated treatment, Scale bar = 100 µm. (F, G) Statistical histogram of Ki‐67 and CXCR4 expression stained with IHC (*n* = 5 per group). Data are presented as mean ± SD. ^*^ *p* < 0.05, ^**^ *p* < 0.01, ^***^ *p* < 0.001. ns: no significance.

As patient‐derived xenografts (PDXs) not only retained original biological characteristics, including cell surface markers and proteomic features, but also maintained tumor heterogeneity and stromal architecture, they exhibit great promise for preclinical applications in drug response prediction and personalized treatment. Therefore, PDX models were employed to improve the clinical translational significance of our findings. The expression of protein biomarkers of ovarian cancer, including paired box gene 8 (PAX8) and P53, was characterized in PDX samples and parental tumors. As shown in Figure S7 and Table S5, the PDXs retained the p53 and PAX8 expression status of their corresponding parental tumors. The expression of CXCR4 in four PDX models was also verified with IHC staining (Figure S8). According to CXCR4 expression levels, these four samples were divided into the CXCR4 low expression group (*n* = 2) and the CXCR4 high expression group (*n* = 2), which were selected for follow‐up experiments (Figure 6A). For [^68^Ga]Ga‐Pentixafor PET imaging, quantitative data from ROI analysis showed that the tumor uptake of [^68^Ga]Ga‐Pentixafor in CXCR4‐high PDX models was much higher than that in CXCR4‐low PDX models (Figure 6B), which was consistent with the expression of CXCR4 with IHC staining.

Next, we evaluated the efficacy of the combination of AMD3100 and CDDP in a PDX model of tumor growth. The combination of AMD3100 and CDDP exerted a more pronounced inhibitory effect on tumor growth than CDDP alone in CXCR4‐high PDX tumors, as evidenced by tumor growth curves and decreased tumor volume and weights. In contrast, the efficacy of these drugs in CXCR4‐low PDX tumors was compromised, as indicated by tumor volume and weight (Figure 6C,D). In line with these findings, the combination of AMD3100 and CDDP in CXCR4‐high expressed groups dramatically diminished the expression of Ki‐67, while the impact on CXCR4‐low expressed groups was less effective (Figure 6E,F). Together, targeting CXCR4 combined with CDDP may be considered a therapeutic strategy for CXCR4‐high‐expressing ovarian cancer patients, guided by [^68^Ga]Ga‐Pentixafor PET imaging for preclinical treatment.

## 3 Discussion

Accumulating studies have demonstrated that CSCs fulfill an essential role in cancer aggressive progression, drug resistance, and tumor relapse in ovarian cancer [39, 40, 41]. To date, therapeutic strategies that can ablate CSCs, relying on the ability to identify and target CSCs, remain a considerable challenge. In this study, we provide functional and translational evidence supporting CXCR4 as a key regulator of ovarian CSC stemness and chemoresistance, and we explore its potential as a theranostic target. Transcriptomic analyses further suggest that CXCR4 upregulation coincides with activation of the NF‐κB/IL‐17/TNF inflammatory pathways, which may contribute to CDDP resistance. While these findings provide correlative mechanistic insights, further studies will be required to delineate the direct causal contributions of these pathways to CXCR4‐mediated chemoresistance. Although AMD3100 monotherapy exhibited weak antitumor activity (see Figure 4F–H), inhibition of CXCR4 with AMD3100 could impair sphere formation and enhance CDDP sensitivity in ovarian CSCs, suggesting that CXCR4‐high cells represent a potential target for a CSC‐directed therapeutic strategy. Significantly, we extended these findings by incorporating CXCR4‐targeted molecular imaging into a precision treatment paradigm. CXCR4‐PET provides real‐time, noninvasive visualization and quantification of stem‐like tumor burden, enabling accurate adjustment of the AMD3100 dose and schedule. Integration of this imaging readout into clinical protocols (CDDP + AMD3100) may counteract the relapse and chemoresistance mediated by CXCR4‐high ovarian CSCs.

Molecular biomarkers and regulators that dictate stemness and self‐renewal properties were needed to guide future strategies for the treatment of ovarian cancer [39]. Existing reports suggest that CXCR4 has long been regarded as a desirable target due to its elevated expression in various malignant tumors [42, 43]. Moreover, CXCR4 was imperative for vascularization, cell proliferation, metastasis, and tumor relapse, which is associated with poor prognosis. A previous study identified CXCR4 as a functional CSC marker for maintaining stemness and regulating tumorigenesis and chemoresistance in lung cancer [44]. CXCR4^+^ CSCs were associated with tumor formation and ascites accumulation, serving as a tool for patient stratification [45]. CXCL12‐CXCR4 axis induced CD133^+^ glioma CSCs to secrete VEGF, thereby promoting the growth and angiogenesis of glioma [46]. In the present study, we demonstrated that CXCR4 was upregulated in spheres derived from ovarian cancer cell lines and could sustain CSC‐like characteristics, including increased self‐renewal and tumor‐initiating capacity, in vitro and in vivo. Our data strengthen those findings from earlier studies showing that high expression of CXCR4 has been implicated in maintaining mesenchymal characteristics of ovarian cancer [25]. While sphere formation and limiting‐dilution assays support the CSC‐like characteristics of CXCR4‐positive cells, we recognize that further validation through serial passaging and long‐term self‐renewal assays is warranted and will be undertaken in future studies. Collectively, our results showed that CXCR4 can be regarded as a CSC marker in ovarian cancer, and that directly targeting CXCR4 may be an effective strategy to eliminate CSCs and achieve tumor regression.

Therapeutic strategies should target these drug‐resistant cell populations to prevent recurrence and refractory disease. Along these lines, CXCR4 inhibitors have attracted attention for their potential use in the treatment of hematological and solid malignancies [43]. It is reported that AMD3100 could enhance the efficacy of chemotherapy in cervical cancer [47] and sensitize prostate cancer cells to docetaxel chemotherapy [48]. Moreover, CXCR4 inhibition could be more potent in combination with a variety of available drugs to resensitize CSCs that are particularly resistant to chemotherapy agents. Previous study showed that a combination of AMD3100 with low‐dose paclitaxel improved the antiproliferative effect of ovarian cancer [29]. Combining AMD3100 with bortezomib could overcome drug resistance in relapsed / refractory multiple myeloma [49, 50]. Moreover, a phase I clinical trial demonstrated that the combination of AMD3100 and bevacizumab increased chemotherapy responsiveness in recurrent high‐grade glioma [51]. In the present research, high expression of CXCR4 in ovarian cancer patients was associated with chemoresistance and tumor relapse. A combination of AMD3100 and CDDP could dramatically suppress sphere formation of ovarian CSCs and inhibit ovarian cancer tumor growth. The relevance of our findings was further supported by a study demonstrating that CXCR4 blockade could inhibit ovarian cancer progression through targeted therapy of ovarian CSCs [52]. Therefore, targeting CXCR4 is an attractive therapeutic strategy for stem cells in ovarian cancer.

Surprisingly, CXCR4‐low tumors remained refractory to AMD3100, even though cytotoxic regimens are typically expected to enrich for CXCR4‐positive, chemoresistant cells. These findings suggest that CXCR4 expression regulation and its role in tumor therapeutic response may be more complex than previously understood. CXCR4 expression may be regulated by multiple factors within the tumor microenvironment, masking or suppressing the induction effects of chemotherapy. Time‐course studies in mice and patients show that doxorubicin or cyclophosphamide can double membrane CXCR4 within 48 h [53], and stromal SDF‐1 then shelters these cells. Furthermore, tumors with low CXCR4 expression may rely on other signaling pathways to maintain survival and drug resistance. Pathways such as PI3K/AKT and Wnt/β‐catenin are closely associated with chemotherapy resistance in multiple tumors [6]. These alternative survival mechanisms may render tumor cells insensitive to CXCR4 blockade, explaining the lack of efficacy of AMD3100 in tumors with low CXCR4 expression.

While AMD3100 is an FDA‐approved CXCR4 antagonist with a well‐established safety record in healthy donors, its combination with cisplatin needs careful evaluation of off‐target effects on normal stem compartments. In the present study, mice receiving the AMD3100–cisplatin regimen exhibited no weight loss or overt organ toxicity. Nevertheless, CXCR4 is physiologically expressed by hematopoietic, mesenchymal, and epithelial stem cells, and chronic blockade could theoretically disturb niche retention or regenerative capacity. A prior study showed that short‐term exposure to AMD3100 significantly reduced the ability of donor‐derived spermatogonial stem cells to establish spermatogenic colonies in recipient mice, without impairing stem cell self‐renewal in vitro, suggesting a therapeutic window [54]. However, this selectivity may be context‐dependent, and prolonged treatment might still compromise CXCR4‐high normal tissues such as the bone marrow or gastric epithelium. Future work should therefore map CXCR4 expression in relevant normal stem cell niches, optimize dosing schedules to allow niche recovery, and incorporate functional assays of long‐term hematopoiesis and mucosal integrity before clinical translation.

Given the significance of CXCR4 in maintaining the stemness of ovarian CSCs and the potential to enhance the antitumor effect of CDDP by targeting CXCR4, determining and visualizing CXCR4 expression levels in vivo was pivotal for therapeutic planning and decision‐making in ovarian cancer. A CXCR4‐targeting radiotracer, [^68^Ga]Ga‐Pentixafor, which has been characterized as highly sensitive and specific for detecting CXCR4 expression noninvasively in vivo, has gained wide attention in PET oncology [32]. Studies showed that [^68^Ga]Ga‐Pentixafor PET/CT imaging has been employed to evaluate the disease burden of CXCR4 receptors in multiple hematological and solid malignancies [55]. [^68^Ga]Ga‐Pentixafor PET/CT imaging provided high contrast images for the detection of CXCR4 expression in vivo in recurrent glioma [56]. All subtypes of lung cancer showed increased uptake of [^68^Ga]Ga‐Pentixafor tracer in the primary lung lesions, indicating high expression of CXCR4 in tumor sites [36]. Our research showed that [^68^Ga]Ga‐Pentixafor PET/CT could qualify SK‐3rd tumors and CXCR4‐high PDX models, and the PET signal correlated well with CXCR4 expression, as confirmed by IHC. CXCR4‐high tumor models selected by [^68^Ga]Ga‐Pentixafor PET/CT are likely to benefit from CXCR4‐targeted therapy. Indeed, our study demonstrated that the CXCR4 inhibitor AMD3100 significantly enhanced the treatment efficacy of CDDP in SK‐3rd tumors and CXCR4‐high PDX models, providing strong support for targeting CXCR4‐high CSC‐driven ovarian cancer through the combination treatment of AMD3100 and CDDP as a novel therapeutic strategy. Compared with prior reports mainly describing CXCR4 overexpression or pharmacologic inhibition, our study combines functional CSC validation, CXCR4‐targeted PET imaging, and therapeutic stratification in patient‐derived models. This integrated approach provides a new translational link between CSC biology and clinical management. Our findings suggest that [^68^Ga]Ga‐Pentixafor PET/CT can guide effective therapy of AMD3100 in combination with standard of care and potentially translate into clinical benefit for ovarian cancer patients who are undergoing chemoresistance and tumor relapse.

## 4 Conclusion

In this study, we demonstrate that disease progression and drug resistance in ovarian cancer frequently coincide with elevated CXCR4 expression. From chemo‐refractory tumors, we isolated the stem‐like SK‐3rd subpopulation. Loss‐of‐function experiments revealed that ovarian CSCs upregulate CXCR4 expression to sustain stemness and CDDP resistance, activating the NF‐κB/IL‐17/TNF inflammatory signaling pathways. These findings support CXCR4 as a tractable therapeutic target for ovarian cancer. Building on these findings, [^68^Ga]Ga‐Pentixafor has been validated as a robust CXCR4‐targeted PET radiotracer for mapping high CXCR4 expression in ovarian cancer, thereby guiding the rational design of CDDP and AMD3100 combination regimens and improving therapeutic efficacy.

## 5 Experimental Section

### 5.1 Cell Lines and Cell Culture

Human ovarian cancer cells SKOV3 (adenocarcinoma, RRID: CVCL_0532), ES‐2 (clear cell carcinoma, RRID: CRL_1978), and murine ovarian epithelial cancer ID8 (RRID: CVCL_IU14) were obtained from the China Center for Type Culture Collection (Wuhan University, Wuhan, China). SK‐3rd (a cisplatin‐resistant subline of SKOV3) was established in our work. The above cell lines were cultured in DMEM/F12 medium supplemented with 10% fetal bovine serum (FBS, Excell Bio, Shanghai, China) in a humidified incubator with 5% CO2 at 37°C. All cell lines used in the experiments were authenticated by short tandem repeat (STR) profiling, which confirmed the absence of cross‐contamination (see the supporting information for the STR reports).

### 5.2 Sphere Formation Assay

Spheres were generated from cells suspension (1000 cells/well) cultured in ultra‐low attachment six‐well plates (Corning, NY, USA) with serum‐free DMEM/F12 medium, containing 2% B‐27 Supplement (Invitrogen, Carlsbad, CA, USA), 20 ng/mL epidermal growth factor (EGF, Peprotech), 20 ng/mL basic fibroblast growth factor (FGF, Peprotech, Rocky Hill, NJ, USA), 10 ng/mL leukemia inhibitory factor (LIF, Peprotech) and insulin‐transferrinselenium (ITS, Invitrogen). After approximately 1–2 weeks of cultivation, images of primary spheres were captured by an inverted fluorescence microscope, and the spheres were harvested, centrifuged, and then dissociated for the following experiment. To analyze the effect of AMD3100 on sphere formation, vehicle and AMD3100 (100 µM) were added to the culture medium for 24 h, and the morphology was observed after 5–10 days in culture.

### 5.3 Establishment of Ovarian Cancer Stem‐Like Cells SK–3rd

The chemoresistant SK‐3rd cell lines were established as previously described [10]. Briefly, 6.0 × 10^6^ SKOV3 cells were subcutaneously injected into the right side of 4‐week‐old female BALB/c‐nu mice (Beijing Vital River, China). Mice were treated with CDDP every other day (3 mg/kg in 0.9% sterile saline; Qilu Pharmaceutical Company, China) intratumorally when the tumor diameter reached approximately 0.5 cm. The control group was injected with an identical volume of 0.9% sterile saline intratumorally. When the solid tumor reached ∼1.5 cm in diameter, single‐cell suspensions were collected by collagenase digestion. Dissociated cells treated with CDDP were cultured in suspension, and the control cells were cultured in adherence. Approximately two weeks later, the above two groups of cells were subcutaneously implanted into BALB/c‐nude mice that were treated intratumorally with CDDP or 0.9% sterile saline, respectively. Freshly isolated tumor cells obtained from the first‐, second‐, and third‐generation xenografts treated with CDDP were named SK‐1st, SK‐2nd, and SK‐3rd cells, respectively, and the control cells obtained from xenografts treated with 0.9% sterile saline were named SK‐NS cells.

### 5.4 Next‐generation mRNA Sequencing

RNA sequencing was conducted by Novogene Technology Co., Ltd. (Wuhan, China). Briefly, SK‐NS and SK‐3rd cells were employed for total RNA extraction using the RNA Nano 6000 Assay Kit of the Bioanalyzer 2100 system (Agilent Technologies, CA, USA). Next, the extracted RNA samples were utilized for reverse transcription, and the cDNA fragments were purified using AMPure XP beads. RNA sequencing was conducted using the Illumina NovaSeq 6000. Differentially expressed genes (DEGs) of the two groups were analyzed by the DESeq2 R package (1.20.0). The *p*‐values were adjusted using Benjamini and Hochberg's method to control the false discovery rate. An adj *p*‐value cutoff of 0.05 and a fold‐change cutoff of 2 were used to define genes with significantly differential expression. Gene Ontology (GO) analysis and Kyoto Encyclopedia of Genes and Genomes (KEGG) enrichment analysis for DEGs were performed using the clusterProfiler R package. Gene Set Enrichment Analysis (GSEA) was performed in RStudio.

### 5.5 IHC Staining

All studies involving human samples were conducted in accordance with the principles of the Declaration of Helsinki, were approved by the Ethics Committee of Tongji Medical College, Huazhong University of Science and Technology (2020S357), and obtained informed consent from all patients. The CXCR4 expression levels were detected in 98 paraffin‐embedded samples obtained from our hospital from 2008 to 2020. The pathological characteristics of these ovarian cancer patients were summarized in Table S1. Immunohistochemical staining of paraffin‐embedded tumor xenografts was used to detect Ki‐67, Cleaved Caspase‐3, p‐H2AX, CXCR4, p53, and PAX8 expression. IHC was performed as follows using a rabbit/mouse enhanced polymer assay system (PV9000, ZSGB‐BIO, China). Briefly, paraffin‐embedded tissue sections were melted, deparaffinized, and rehydrated, then antigen‐retrieved in citrate buffer (pH 6). Sections were treated with hydrogen peroxide to block endogenous peroxidase and incubated with primary antibodies at 4°C overnight. Information on primary antibodies is listed in Table S6. Next, the sections were incubated with reaction enhancement solution at 37°C for 20 min. After washing with PBS three times, the enhanced enzyme‐labeled anti‐rabbit or mouse IgG was added and incubated for 30 min at 37°C. Finally, sections were visualized with diaminobenzidine and counterstained with hematoxylin. Images were taken by a microscope (Olympus IX71, Olympus, Japan).

### 5.6 Primary Ovarian Cancer Cells Isolation

All studies involving tumor tissues were surgically resected and approved by the Ethics Committee of Tongji Medical College, Huazhong University of Science and Technology (IORG0003571). Fresh tumor mass was obtained from clear surgical fields, cut into approximately 1 mm^3^ pieces, and then enzymatically digested with a collagenase solution. Following filtration by a 70 µm Cell‐Strainer (BD, USA), the cell suspension was neutralized by an equal volume of complete medium and pelleted at 300 × *g*. After removing red blood cells with lysis buffer (Solarbio, CHN), the filtered cells were eventually resuspended in PBS for the following experiments.

### 5.7 Flow Cytometry

Different stem cell markers (ALDH) and CXCR4 in ovarian cancer cells were detected using flow cytometry (BD LSRFortessa X‐20, USA). In brief, cells were resuspended in PBS and stained with PE/CY7‐anti‐CXCR4 at a dilution of 1:500 (Biolegend, 306514) for 30 min at 4°C. The ALDEFLUOR Assay Kit (#01700; Stemcell Technologies) was used to detect ALDH activity according to the manufacturer's instructions. The ALDH^+^ population was identified with flow cytometry. Details of gating strategies are schematically illustrated in the supplementary figure, including forward/side‐scatter exclusion, doublet removal, and negative controls for CXCR4 and ALDH staining (Figure S9). Data were analyzed using FlowJo software (10.8.1).

### 5.8 In Vivo Limited Dilution and Tumorigenesis Assay

CXCR4‐knockdown cell lines (SK‐3rd‐shCXCR4 and ID8‐shCXCR4) were generated using the CRISPR/Cas9 method. BALB/c‐nu/nu mice were randomized and subjected to in vivo limiting‐dilution assays by subcutaneous injection of defined cell numbers; controls received vector‐only cells. Tumor volume and body weight were measured with calipers every 48 h. Animals were euthanized when the longest tumor axis approached 20 mm. Group identity was concealed from the investigator during data collection.

### 5.9 Bioinformatics Analysis

The mRNA expression of CXCR4 was analyzed using the TCGA‐OV database (https://portal.gdc.cancer.gov/) and the GTEx database (https://xenabrowser.net/datapages/). GSE15372 (5 sensitive and 5 resistant cells) was used to explore CXCR4 expression in the platinum‐sensitive and resistant groups, after normalization [57]. The prognostic value of CXCR4 in ovarian cancer was analyzed using the Kaplan‐Meier plotter (http://kmplot.com) and the GSE30161 database [58]. GEPIA (http://gepia.cancer‐pku.cn/) analysis was conducted to examine the correlation of CXCR4 with multiple stem cell markers.

### 5.10 scRNA‐seq Data Analysis

The dataset GSE158722 was downloaded from the GEO database (https://www.ncbi.nlm.nih.gov/geo/) [59]. Eight pre‐treatment and six post‐treatment malignant ascites or pleural samples were selected for the scRNA‐seq analysis. The gene expression matrix was converted to Seurat objects using the Seurat R package (version 4.2.2). Cells were removed when the number of detected genes was less than 500, and the top 2000 highly variable genes were selected for further clustering analysis. After scaling, the data were normalized, and PCA was performed on the highly variable genes using the RunPCA function. Cells were clustered using the FindNeighbors and FindClusters functions with a resolution of 0.1. The first 20 principal components were selected to reduce dimensionality further using UMAP. Then, the distribution of ovarian cancer stem cell markers ALDH1A1, CD133, and CXCR4 in pre‐ and post‐treatment samples was analyzed, and all analyses and visualizations were completed using RStudio (version 4.1.0).

### 5.11 Cell Viability Assay

Approximately 5 × 10^3^ cells/well (SK‐3rd cells and ES‐2 cells) were plated in 96‐well plates overnight and then treated with the indicated concentrations of CDDP and AMD3100. Cellular viability was determined using a (3‐(4,5‐dimethylthiazol‐2‐yl)‐2,5‐diphenyltetrazolium bromide) (MTT) (Aladdin, Shanghai, China) according to the manufacturer's instructions at the indicated time points. Briefly, the plates were treated with 20 µL of MTT solution (5 mg/mL in phosphate‐buffered saline) and incubated for another 4 h at 37°C. After being dissolved in 150 µL dimethyl sulfoxide per well, the plates were shaken and measured at 570 nm with a microplate reader (SpectraMax, Sunnyvale, CA, USA). The synergistic effect of the drug combination was determined using SynergyFinder software (https://synergyfinder.fimm.fi), and the zero‐interaction potency (ZIP) model was used to calculate the drug interaction. Red regions with ZIP scores > 10 indicated synergism (a white frame marks the regions of highest synergy), whereas the green regions with ZIP < −10 indicated antagonism.

### 5.12 Colony Formation Assay

SK‐3rd cells and ES‐2 cells were seeded into 6‐well plates at a density of 10^3^ cells per well. Cells were treated with CDDP and AMD3100 at the indicated concentrations for 48 h, then cultured in fresh complete medium. After the experiments were terminated, colonies were fixed with 4% paraformaldehyde for 30 min and stained with 0.1% crystal violet for 1 h. Finally, the clone numbers were counted using ImageJ.

### 5.13 qRT‐PCR

Total cellular RNA was extracted using TRIzol reagent (TaKaRa, Japan), and reverse transcription into cDNA was performed with the HiScriptIII RT SuperMix kit (Vazyme, China) according to the manufacturer's instructions. qRT‐PCR was performed using AceQ qPCR SYBR Green Master Mix (Vazyme, China). *β‐ACTIN* served as a reference gene to normalize target gene expression levels. The relative abundance of the genes was determined using the 2^−ΔΔCt^ method. The primer sequence for each gene is listed in Table S7.

### 5.14 [^68^Ga]Ga‑Pentixafor and [^68^Ga]Ga‑FAPI‐04 Radiolabeling

The Pentixafor and FAPI‐04 were purchased from MedChemExpress (Monmouth Junction, NJ, USA). ^68^Ga was eluted from a ^68^Ge/^68^Ga generator (GalliaPharm, Eckert and Ziegler Radiopharma GmbH). The labeling with ^68^Ga was carried out according to a published method [60]. Briefly, Pentixafor and FAPI‐04 were dissolved in a mixture of ^68^GaCl_3_ and 0.1 M sodium acetate buffer adjusted to pH 4.0, then purified using C18 cartridges after incubating the reaction solution at 95 degrees for 15 min. The radiochemical yield and radiochemical purity were determined using radio high‐performance liquid chromatography (Shimadzu, Kyoto, Japan).

### 5.15 PET/CT Imaging in Tumor‐Bearing Mice

PET/CT imaging was conducted with [^68^Ga]Ga‐FAPI‐04 and [^68^Ga]Ga‐Pentixafor, respectively. Xenograft mice bearing SK‐NS and SK‐3rd cells were scanned using a small animal PET/CT scanner (RAYCAN Technology Co, Ltd., Suzhou, China). For the subcutaneous xenograft model bearing SK‐NS and SK‐3rd cells (*n* = 3 per group), about 3.7 to 7.4 MBq of [^68^Ga]Ga‑Pentixafor and [^68^Ga]Ga‐FAPI‐04 were administered intravenously, respectively. PET/CT imaging was performed under isoflurane anesthesia at 15, 30, 60, and 120 min post‐injection. Acquisition time for each scan was approximately 15 min. Biodistribution studies were performed at the corresponding time points after intravenous injection. Quantitative results were obtained by drawing regions of interest (ROIs) in the tumor and tissue organs, and the data were analyzed in Inveon Research Workspace (Siemens).

### 5.16 Subcutaneous Xenograft Model

Four‐week‐old female BALB/c‐nude mice were obtained from Vital River Laboratory Animal Technology (Beijing, China) and kept in a specific pathogen‐free (SPF) husbandry environment. All animal experiments were approved by the Institutional Animal Care and Use Committee of Wuhan Youdu Biological Technology (No. 20221226).

For tumorigenicity experiments, SK‐3rd cells (10^4^ and 10^5^ cells) and SK‐NS cells (10^4^ and 10^5^ cells) were subcutaneously injected into female BALB/c‐nude mice, respectively. For evaluating the effect of CXCR4 on tumorigenicity, CXCR4^+^ cells and CXCR4^−^ cells were sorted from ES‐2 cells by flow cytometry, and then CXCR4^+^ (10^3^ and 10^4^ cells) and CXCR4^−^ (10^3^ and 10^4^ cells) were implanted subcutaneously into BALB/c‐nude mice, respectively. On the indicated dates after cell inoculation, the mice were sacrificed when the tumor length was < 20 mm, and the number of tumors was counted.

For evaluation of antitumor efficacy, a total of 10^6^ SK‐3rd cells and ES‐2 cells were subcutaneously inoculated into the right flanks of the mice, respectively. When the tumor volume (*V*) reached approximately 50–100 mm^3^, mice were randomly assigned into four groups (*n* = 5 per group) and intraperitoneally treated with vehicle, AMD3100 (3 mg/kg every 2 days), CDDP (3 mg/kg every 3 days), and AMD3100 + CDDP for 2 weeks. Tumor volume and body weight were monitored every two days. Mice were sacrificed at the end of the experiment with a maximal tumor length of < 20 mm. The tumors were isolated and weighed, and tumor size was calculated as (length× width^2^)/2. The tumor tissues were fixed with 10% formaldehyde for subsequent histological examination. Tumor suppression rate (TSR) (%) was calculated using the following equation: TSR (%) = [(Vc – Vx) / Vc] × 100%, where Vc and Vx indicated the average tumor volume of the control group and treatment group, respectively. The synergistic effect of CDDP and AMD3100 was evaluated as follows: *Q* = *E*_(A + B)_/[*E*_(A)_ + *E*_(B)_ – *E*_(A)_ × *E*_(B)_], where *E*_(A)_, *E*_(B)_, and *E*_(A + B)_ represent the TSRs of the respective groups. *Q* < 0.85, 0.85 ≤ *Q* < 1.15, and *Q* ≥ 1.15 represent antagonistic, additive, and synergistic effects, respectively.

### 5.17 PET/MR Imaging in a Patient with Ovarian Cancer

The diagnostic performance of [^68^Ga]Ga‐FAPI‐04 and [^68^Ga]Ga‐Pentixafor was further evaluated in patients with primary ovarian cancer. The clinical study was approved by the Independent Ethics Committee of Union Hospital, Tongji Medical College, Huazhong University of Science and Technology (UHCT230641). The informed consent form was obtained from the patient with ovarian cancer. All procedures were conducted in accordance with the principles in the Declaration of Helsinki and the ethical guidelines of national research committees. PET/MR were performed using the GE Healthcare SIGNA TOF PET/MRI (3.0 T) model. PET/MR imaging of the whole body and abdominal pelvic cavity was obtained approximately 30 min after intravenous injection of [^68^Ga]Ga‐FAPI‐04 (111 MBq) and [^68^Ga]Ga‐Pentixafor (136 MBq), respectively, with a total scan duration of approximately 45–60 min. The concerned cancer lesions were manually drawn on the serial images, and the radioactivity concentration and maximum standardized uptake value (SUV_max_) within the volumes of interest were automatically obtained from the workstation. The expression of CXCR4 in various tumor sites was assessed by immunohistochemical staining.

### 5.18 Therapeutic Study with PDX Models

Four‐week‐old female NVSG mice were obtained from Beijing View Solid Biotechnology, China, and kept in a SPF husbandry environment. Patient‐derived tumors of ovarian cancer were collected, and the fascia, blood vessels, and necrotic tissue were removed in a biological safety cabinet. The retained tissue was washed with PBS several times and cut into approximately 1 mm^3^ fragments, and then transplanted subcutaneously into NVSG mice. NVSG mice were kept until the tumor size reached 50–100 mm^3^. For PET/CT imaging, [^68^Ga]Ga‑Pentixafor was administered intravenously into NVSG mice, and PET/CT imaging was performed as mentioned above. For evaluation of antitumor efficacy, the mice were randomly assigned to two groups (*n* = 5 per group): CDDP and CDDP + AMD3100. CDDP was administered intraperitoneally (3 mg/kg) every 3 days for 2 weeks, and AMD3100 was administered intraperitoneally (3 mg/kg) every other day for 2 weeks. Body weight and tumor volume were recorded every other day. Mice were humanely euthanized at the end of the experiment with a maximal tumor length < 20 mm. The tumor volume (*V*) was calculated as (length × width^2^)/2. The tumor tissues were fixed with 10% formaldehyde for subsequent immunohistochemical staining and HE staining.

### 5.19 Statistical Analysis

All experiments were independently repeated at least three times, and data are presented as mean ± standard deviation (SD), where n represents the number of independent biological replicates. Comparisons between two groups were performed using a two‐tailed Student's t‐test. One‐way analysis of variance (ANOVA) followed by appropriate post‐hoc multiple comparison tests was used to evaluate differences among multiple groups (*n* > 2). Survival analyses were conducted using the Kaplan–Meier method, and *p* values were calculated using the log‐rank test. All statistical analyses were performed using IBM SPSS v22 (IBM, Armonk, NY, USA) and GraphPad Prism 8 (San Diego, CA, USA). A *p* value < 0.05 was considered statistically significant.

## Author Contributions

Conceptualization: J.C., L.L.G., D.W.J.; Methodology: L.X.F., S.M.Z., Z.W., W.W.W., F.Q.Y., L.H.; Investigation: L.X.F., S.M.Z., Z.W., F.Q.Y., L.H. (patient–derived xenograft model), M.N.Z., M.Q.C. (clinical samples and related analysis); Data Curation: W.W.W. (RNA‐seq analysis), L.X.F., S.M.Z., Z.W.; Formal Analysis: L.X.F., S.M.Z., Z.W., W.W.W.; Resources: F.Q.Y., L.H., M.N.Z., M.Q.C.; Writing – Original Draft: L.X.F., S.M.Z., Z.W.; Writing – Review & Editing: Y.Q., S.S.; Supervision: J.C., L.L.G., D.W.J.; Technical Support: W.W.W.; Project Administration: J.C., L.L.G., D.W.J. All authors read and approved the final manuscript. L.X.F., S.M.Z., and Z.W. contributed equally to this work. Authorship order among the co‐first authors was determined according to their relative contributions.

## Conflicts of Interest

The authors declare no conflict of interest.

## Supporting information




**Supporting File**: advs74106‐sup‐0001‐SuppMat.docx.

## Data Availability

The data that support the findings of this study are available in the supplementary material of this article.
