# Supplementary material for: Mapping CSC‐Mediated Ovarian Cancer Chemoresistance via CXCR4‐PET to Guide Precision Cisplatin Re‐Sensitization Therapy
Source: Adv Sci (Weinh). 2026 Feb 3;13(19):e21279. doi: 10.1002/advs.202521279 (PMC13045449; doi:10.1002/advs.202521279)
Supplement: Supplementary file 1 — Supporting File: advs74106‐sup‐0001‐SuppMat.docx. [file ADVS-13-e21279-s001.docx]

**Mapping CSC-Mediated Ovarian Cancer Chemoresistance via CXCR4-PET to Guide Precision Cisplatin Re-sensitization Therapy**

Lixia Feng^1,2,3,4#^, Simei Zhao^1#^, Zheng Wei^5#^, Wenwen Wang^1^, Feiquan Ying^1^, Lin Huang^1^, Mengna Zhu^1^, Mengqing Chen^1^, Qiang Yang^1^, Si Sun^1^, Dawei Jiang^2,3,4*^, Lingling Gao^1,6*^, Jing Cai^1*^

^1^ Department of Obstetrics and Gynecology, Union Hospital, Tongji Medical College, Huazhong University of Science and Technology, Wuhan 430022, China

^2^ Department of Nuclear Medicine, Union Hospital, Tongji Medical College, Huazhong University of Science and Technology, Wuhan 430022, China

^3^ Hubei Key Laboratory of Molecular Imaging, Wuhan 430022, China

^4^ Key Laboratory of Biological Targeted Therapy, the Ministry of Education, Wuhan 430022, China

^5^ Department of Obstetrics and Gynecology, Third Hospital of Shanxi Medical University, Shanxi Bethune Hospital, Shanxi Academy of Medical Sciences, Tongji Shanxi Hospital, Taiyuan, China

^6^ Department of Obstetrics and Gynecology, Zhongnan Hospital of Wuhan University, Wuhan, 430071, China

^#^ Lixia Feng, Simei Zhao, and Zheng Wei contributed equally to this work.

*Corresponding author:

Dawei Jiang: daweijiang@hust.edu.cn

Simei Zhao: 2017xh0069@hust.edu.cn

Jing Cai: jingcai@hust.edu.cn


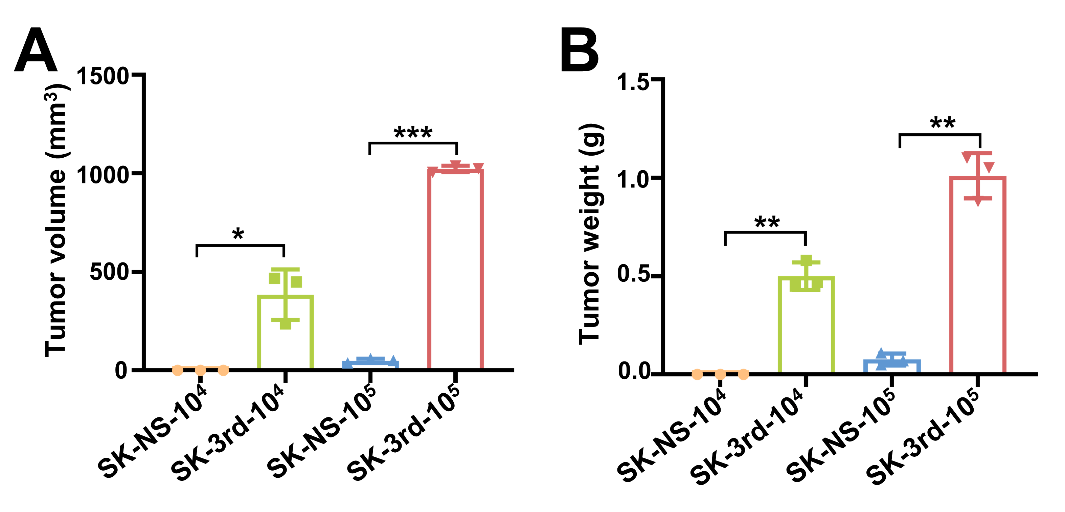


**Figure S1 A, B) The volumes (A) and weights (B) of tumors isolated from the sacrificed mice.** **Data are presented as mean ± SD. **P* < 0.05, ***P* < 0.01, *** *P* < 0.001.**

**
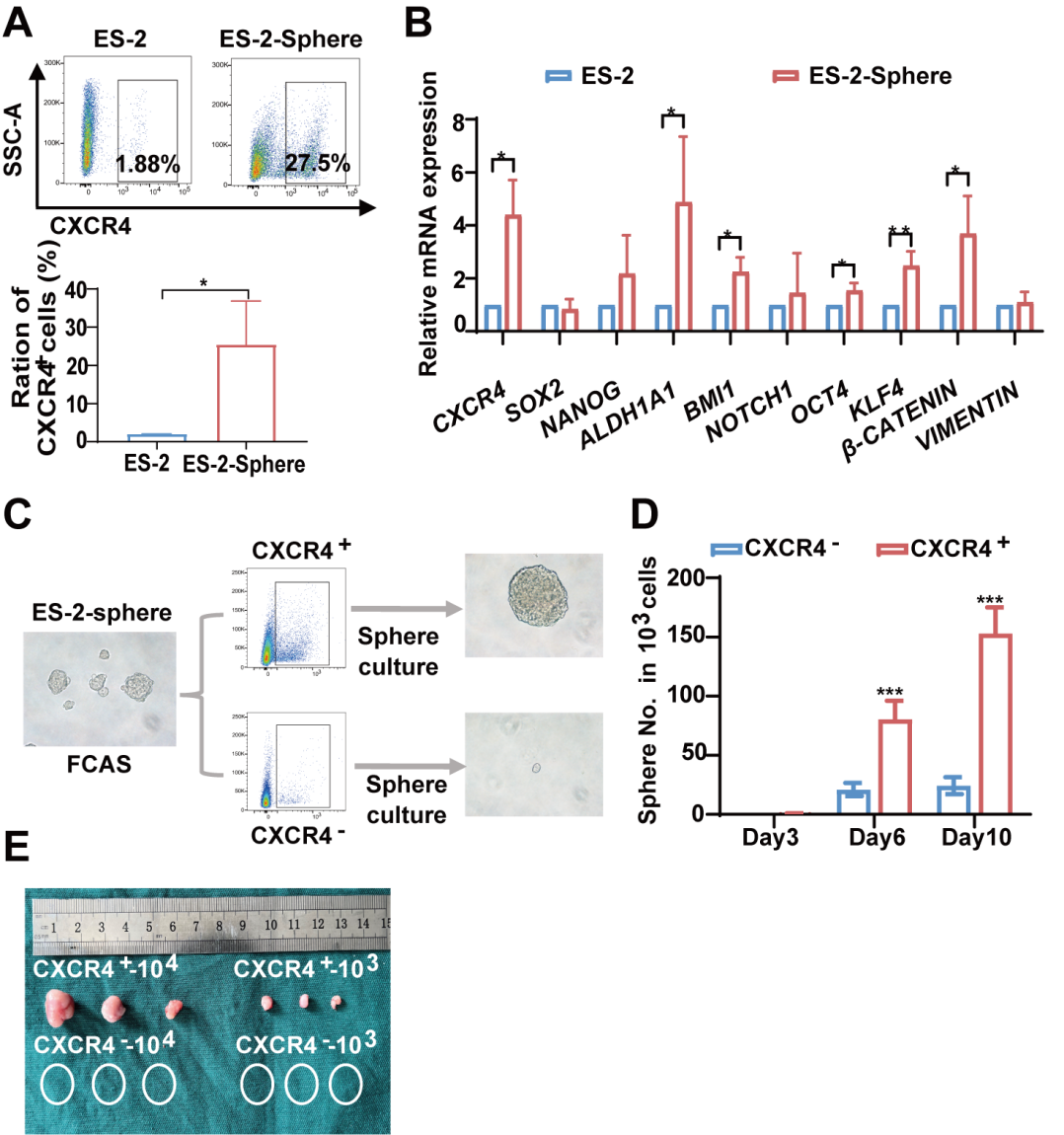
**

**Figure S2. A)** Percentage of CXCR4^+^ cell population in ES-2 cells with adherent and sphere conditions detected by flow cytometry (n = 3). **B)** mRNA level of CSC-associated markers in ES-2 cells with adherent and sphere conditions detected by qRT-PCR (n = 3). **C)** Schematic illustration for sorting CXCR4^-^ and CXCR4^+^ spheres derived from ES-2 cells with FACS. **D)** Numbers of spheres formed by CXCR4^-^ and CXCR4^+^ ES-2 cells on day 3, day 6, and day10, respectively (n = 3). **E)** Tumor-initiating capability of CXCR4^-^ and CXCR^+^ cells examined by xenograft experiment (n = 3 / group). Data are presented as mean ± SD of three independent experiments or triplicates. A two-tailed unpaired t-test determined P values. **P* < 0.05.

**
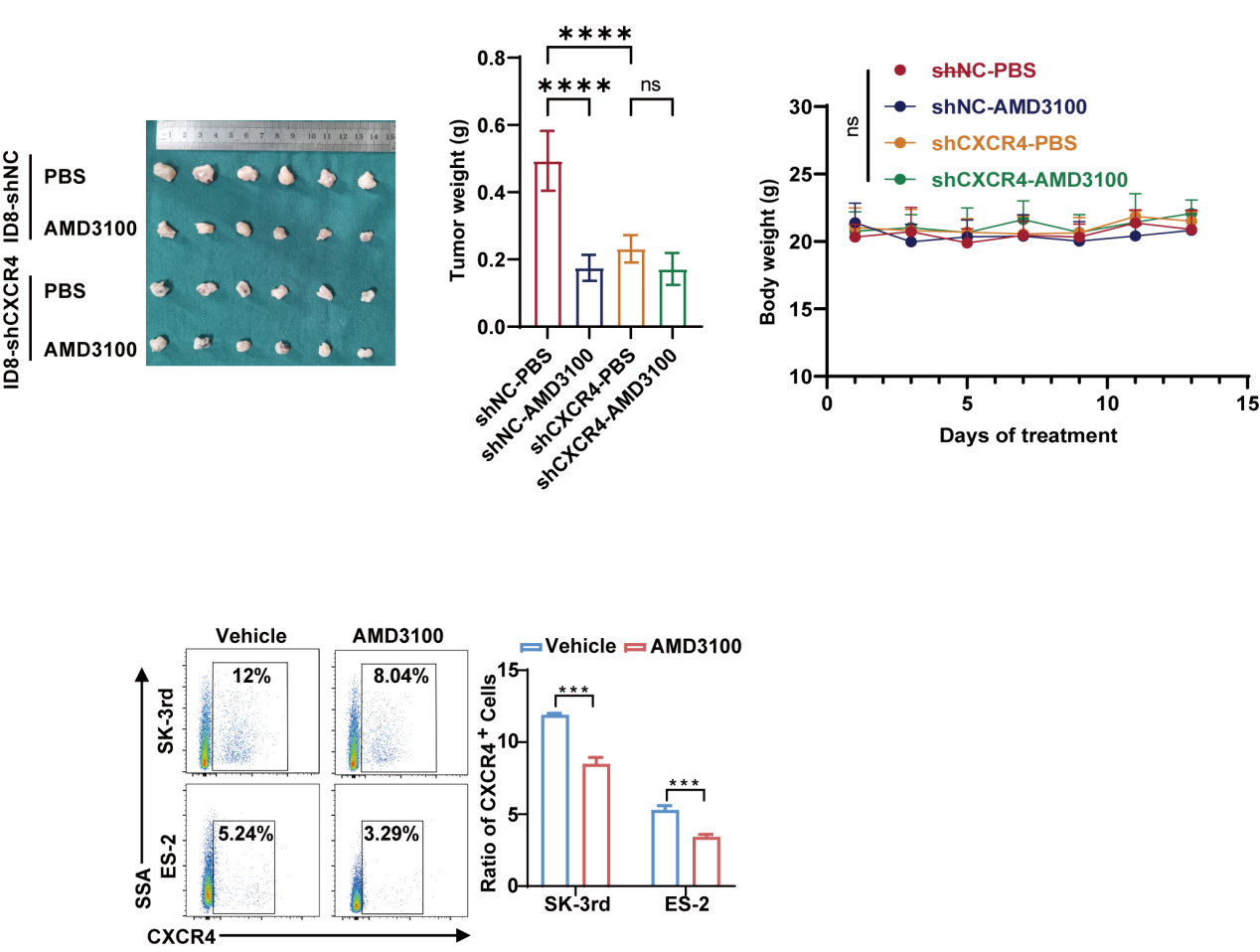
**

**Figure S3.** Ratio of CXCR4^+^ spheres formed by SK-3rd and ES-2 cells treated with vehicle and AMD3100 (100 μM) detected by flow cytometry.

**
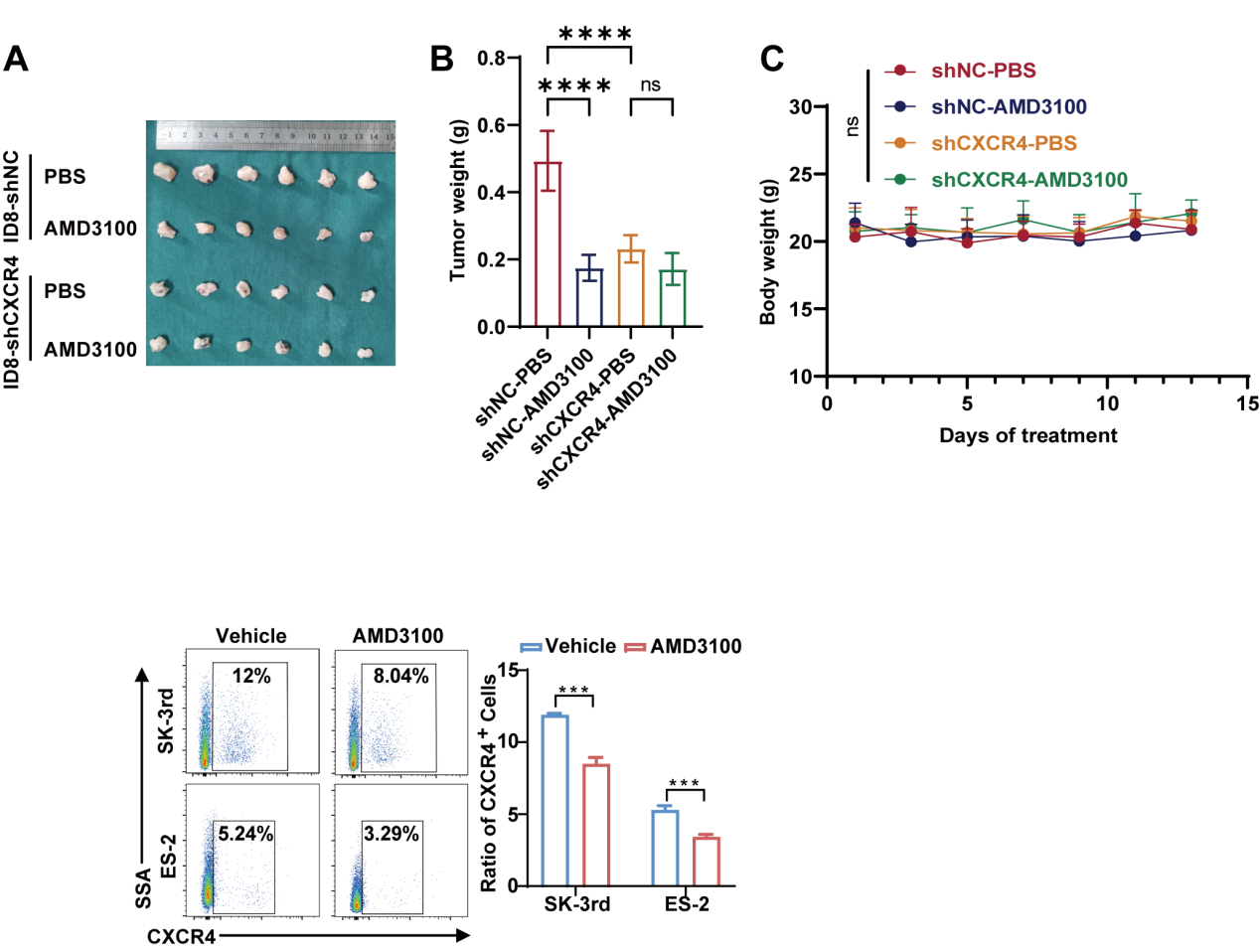
**

**Figure S4. (A) *ex vivo* tumor images, (B) tumor weight, and (C) Body weight of the AMD3100 treatment assay. Data are presented as mean ± SD. *****P* < 0.001**

**
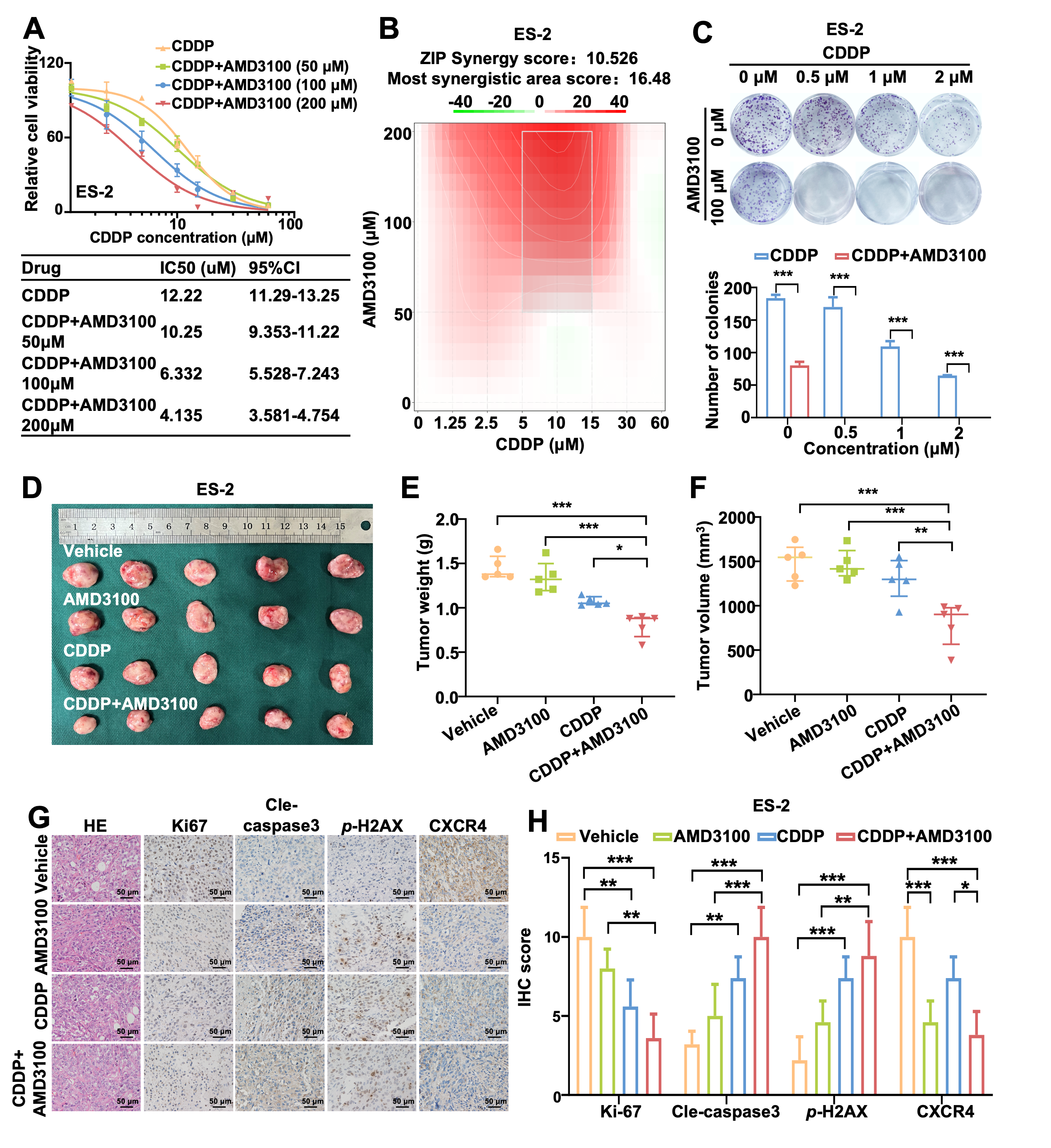
**

**Figure S5. CXCR4 inhibitor AMD3100 enhanced CDDP sensitivity of OC *in vitro* and *in vivo*. A)** Cell viability of ES-2 cells treated with AMD3100 and CDDP with different concentrations detected by MTT assay. **B)** Synergistic cytotoxic effects of AMD3100 and CDDP on ES-2 cells analyzed with SynergyFinder2.0. **C)** Colony formation assay of ES-2 cells treated with AMD3100 (0 μM and100 μM) and CDDP (0 μM, 0.5 μM, 1 μM, and 2 μM). **D)** Represent images of tumors derived from mice bearing ES-2 cells treated with vehicle, CDDP, AMD3100, and CDDP + AMD3100. n = 5 mice for each group. **E, F)** Tumor volume **(E)** and tumor weight **(F)** in mice bearing ES-2 cells treated with vehicle, CDDP, AMD3100, and CDDP + AMD3100. n = 5 mice for each group. **G, H)** H&E staining of tumors and immunohistochemistry for Ki-67, cle-caspase3, *p*-H2AX, and CXCR4 in xenografts from mice treated with vehicle, CDDP, AMD3100, and CDDP + AMD3100. Data are presented as mean ± SD. **P* < 0.05, ***P* < 0.01, *** *P* < 0.001.


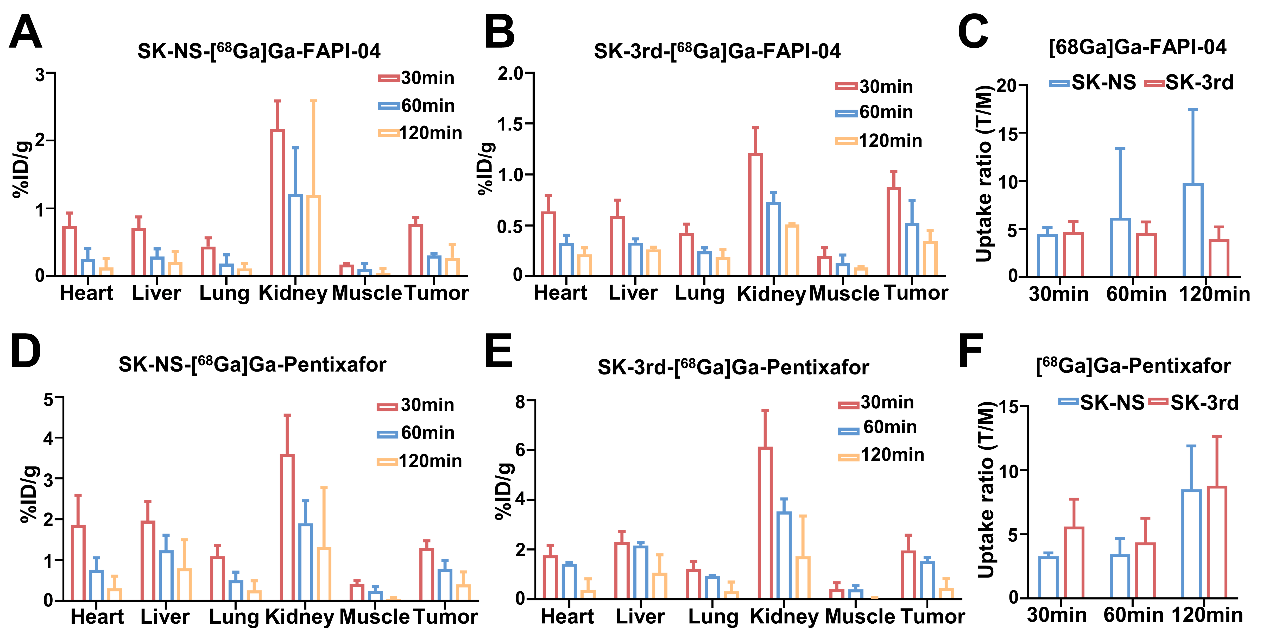


**Figure S6. Biodistribution of SK-NS and SK-3rd tumor-bearing mice over time and tumor-to-muscle(T/M) ratios at indicated times in SK-NS and SK-3rd tumor-bearing mice models.** **(A-B)** Biodistribution of SK-NS tumor-bearing mice **(A)** and SK-3rd tumor-bearing mice **(B)** injected with [^68^Ga]Ga-FAPI-04. **(C-D)** Biodistribution of SK-NS tumor-bearing mice **(C)** and SK-3rd tumor-bearing mice **(D)** injected with [^68^Ga]Ga-Pentixafor. **(E-F)** T/M ratios of [^68^Ga]Ga-FAPI-04 **(E)** and [^68^Ga]Ga-Pentixafor **(F)** at the indicated time points.


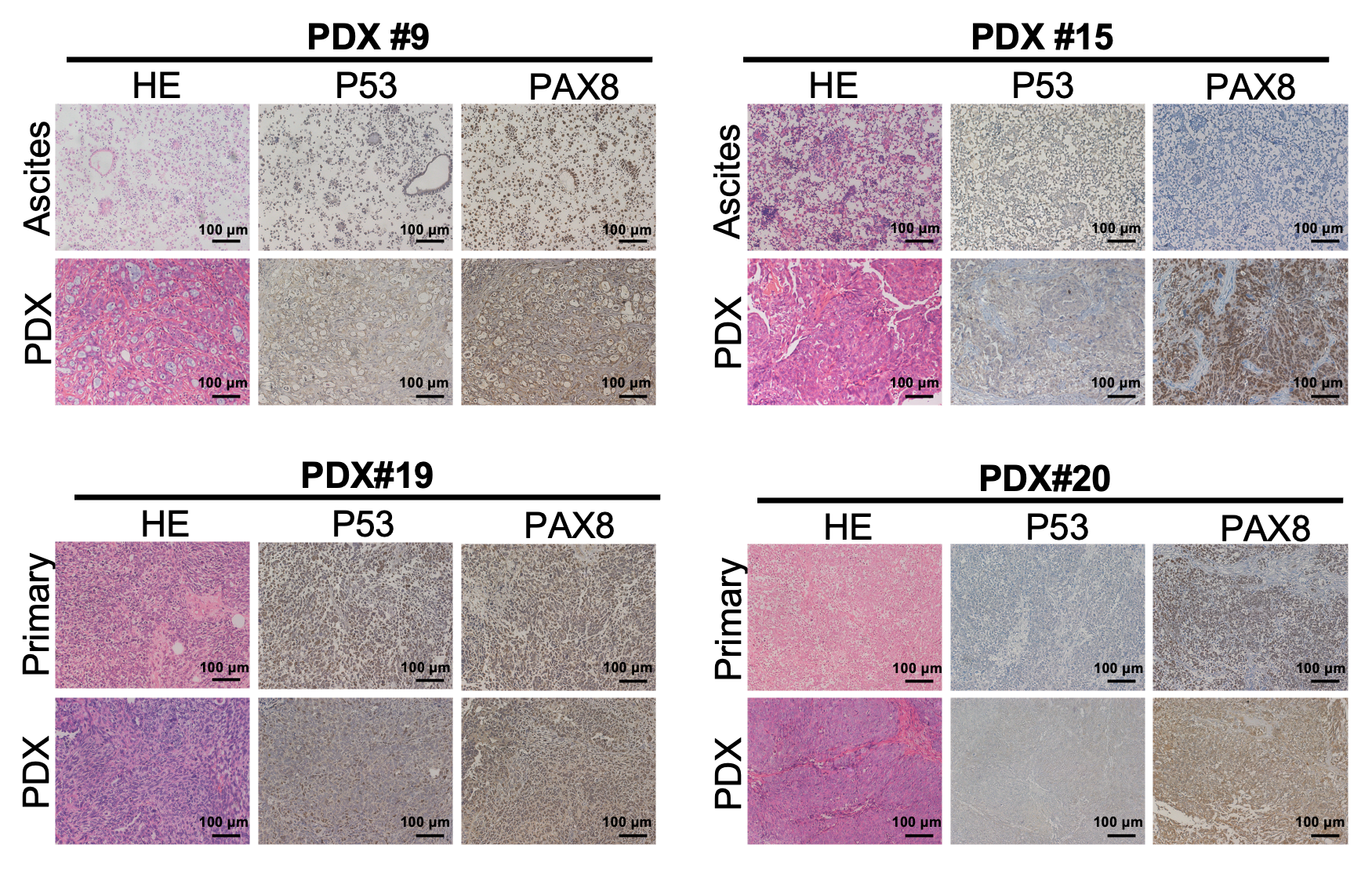


**Figure S7. Phase-contrast images of four PDX with H&E staining (left) and immunohistochemical staining (right) of P53 and PAX8 in PDX samples and corresponding parental tumors.**


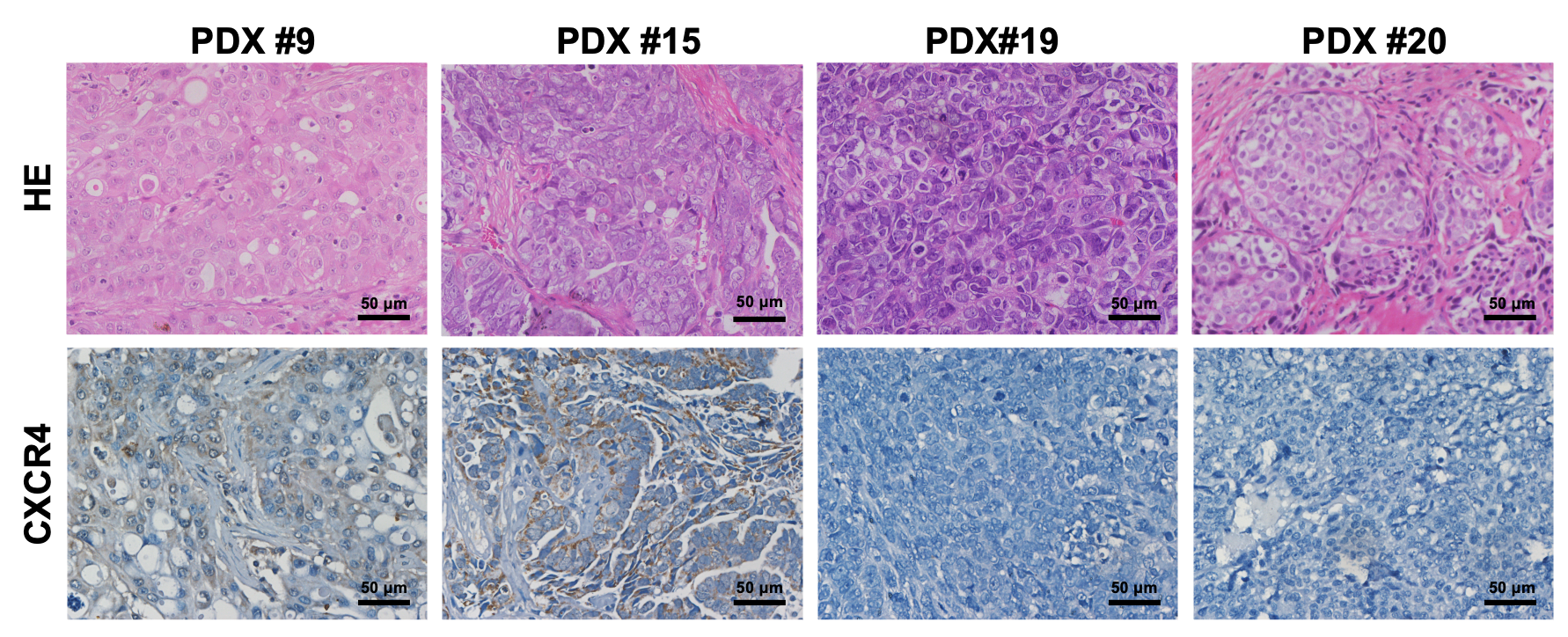


**Figure S8. Represent images of H&E staining (up) and immunohistochemical staining (down) of CXCR4 in four PDX samples.**


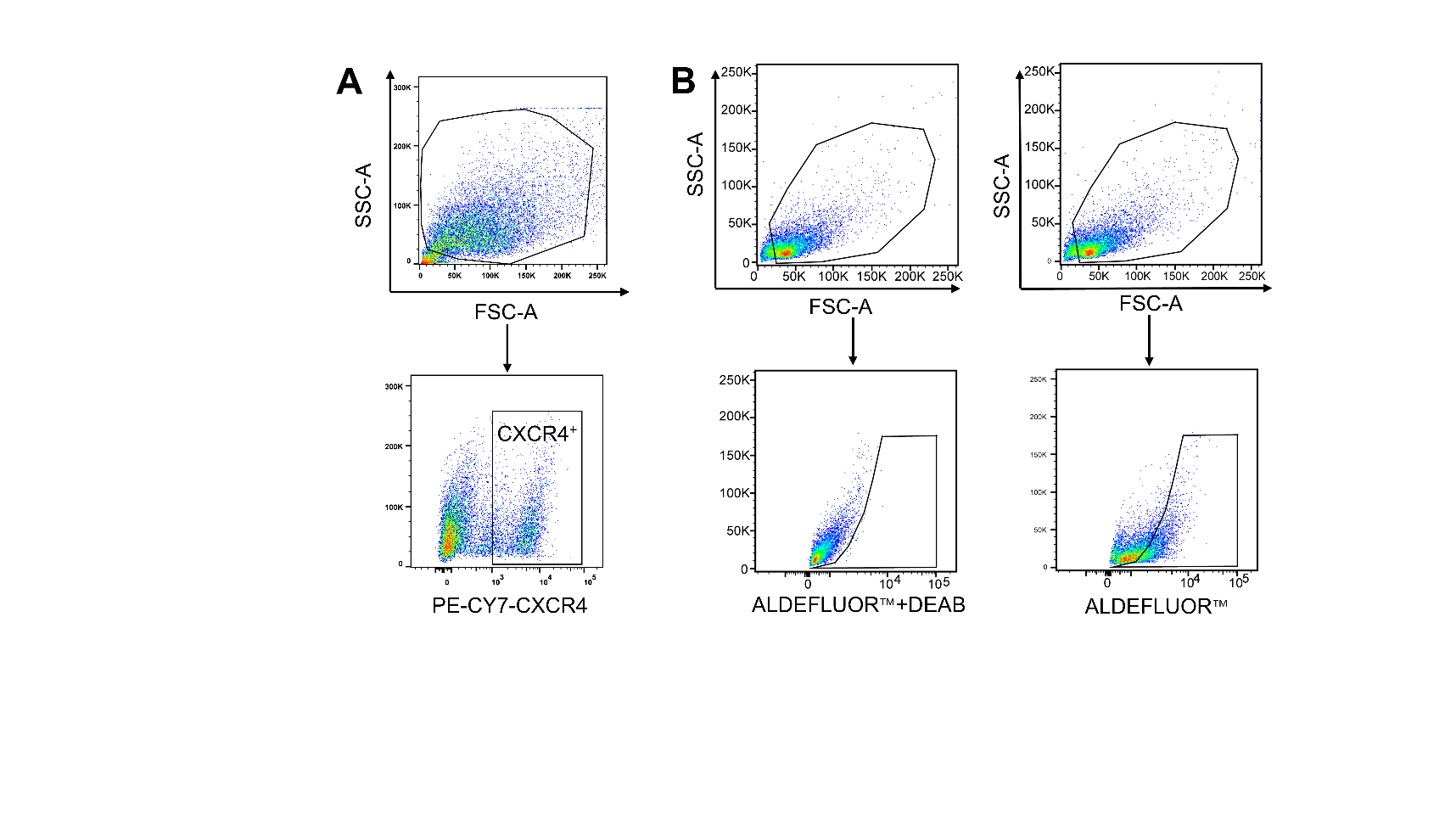


**Figure S9. Scheme for flow sorting and analysis. (A) Cell sorting scheme for CXCR4^+^cells from PDCs and SK-NS and SK-3rd cells performed by FACS. (B) Scheme for ALDH+ cells from SK-NS and SK-3rd cells by flow analysis. ALDEFLUOR^TM^+DEAB represents the negative control, ALDEFLUOR^TM^ represents the ALDH^+^ cells in total cells.**

**Table S1: The correlation of CXCR4 expression with clinicopathological characteristics of OC patients**

| **Clinicopathological parameters** | **Cases (%)** | **CXCR4** | | ***P-*value** |
| --- | --- | --- | --- | --- |
|  |  | **Low (%)** | **High (%)** |  |
| **Age (years)** |  |  |  |  |
| <50 | 36 (36.7%) | 13 (36.1%) | 23 (63.9%) | 0.238 |
| >=50 | 62 (63.3%) | 30 (48.4%) | 32 (51.6%) |  |
| **Histological subtype** |  |  |  |  |
| Serous | 84 (85.7%) | 39 (46.4%) | 45 (53.6%) | 0.213 |
| Others | 14 (14.3%) | 4 (28.6%) | 10 (71.4%) |  |
| **FIGO stage^1^** |  |  |  |  |
| I-II | 24 (24.5%) | 18 (75.0%) | 6 (25.0%) | *P*<0.001 |
| III-IV | 74 (75.5%) | 25 (33.8%) | 49 (66.2%) |  |
| **Chemotherapy response^2^** |  |  |  |  |
| Sensitive | 69 (70.4%) | 38 (55.1%) | 31 (44.9%) | 0.001 |
| Resistant | 29 (29.6%) | 5 (17.2%) | 24 (82.8%) |  |
| **Recurrence** |  |  |  |  |
| No | 36 (36.7%) | 23 (63.9%) | 13 (36.1%) | 0.002 |
| Yes | 62 (63.3%) | 20 (32.3%) | 42 (67.7%) |  |

^1^ FIGO: Federation International of Gynecology and Obstetrics

^2^ Chemotherapy resistance indicates cancers that display no response to chemotherapy (stable disease), progression of disease during chemotherapy, or relapse within 6 months of the completion of primary chemotherapy.

**Table S2** **Clinical information of PDCs**

| **ID** | **Age（years）** | **FIGO stage** | **Neoadjuvant chemotherapy** | **Histological type** | **Tumor site** |
| --- | --- | --- | --- | --- | --- |
| PDC#1 | 57 | IV | No | High-grade serous ovarian cancer | Primary tumor |
| PDC#2 | 67 | IIIC | No | High-grade serous ovarian cancer | Ascites |
| PDC#3 | 47 | IVB | Yes | High-grade serous ovarian cancer | Primary tumor |
| PDC#4 | 43 | IIIC | No | High-grade serous ovarian cancer | Ascites |
| PDC#5 | 57 | IC | No | Endometrioid ovarian cancer | Primary tumor |
| PDC#6 | 57 | IIIC | No | Carcinosarcoma | Primary tumor |

**Table S3 Tumor suppression rate (TSR%) and Q value in SK-3rd tumor-bearing mice treated with different agents on Day 14.**

| Group | PBS | AMD3100 | CDDP | AMD3100+CDDP |
| --- | --- | --- | --- | --- |
| Mean ± SD (mm^3^) | 470.01 ± 104.35 | 416.77 ± 58.31 | 337.63 ± 51.98 | 159.36 ± 94.87 |
| TSR (%) | - | 12.08 | 28.77 | 66.38 |
| Q |  |  |  | 1.78 |

**Table S4 Tumor suppression rate (TSR%) and Q value in ES-2 tumor-bearing mice treated with different agents on Day 14.**

| Group | PBS | AMD3100 | CDDP | AMD3100+CDDP |
| --- | --- | --- | --- | --- |
| Mean ± SD (mm^3^) | 1483.98 ± 205.90 | 1440.23 ± 119.06 | 1306.16 ± 239.06 | 798.14 ± 249.20 |
| TSR (%) | - | 2.95 | 11.98 | 46.22 |
| Q |  |  |  | 3.17 |

**Table S5 Clinical information of patient-derived xenografts (PDXs)**

| **ID** | **Age**  **(years)** | **FIGO stage** | **Neoadjuvant chemotherapy** | **Chemoresistance** | **Histological type** | **Tumor site** |
| --- | --- | --- | --- | --- | --- | --- |
| PDX#9 | 47 | III | No | No | High-grade serous ovarian cancer | Ascites |
| PDX#15 | 42 | IIIC | No | No | High-grade serous ovarian cancer | Ascites |
| PDX#19 | 78 | II | No | No | Carcinosarcoma | Primary tumor |
| PDX#20 | 40 | IIIB | No | No | High-grade serous ovarian cancer | Primary tumor |

**Table S6 List of primary antibodies**

| **Name** | **Catalog** | **Manufacturer** | **Application(s)** |
| --- | --- | --- | --- |
| PE/Cyanine7-CXCR4 | 306514 | BioLegend | FACS |
| CXCR4 | 60042-1-Ig | Proteintech | IHC(1:300） |
| P53 | A0263 | ABclonal | IHC(1:200） |
| PAX8 | 10336-1-AP | Proteintech | IHC(1:500） |
| Ki-67 | 27309-1-AP | Proteintech | IHC(1:3000） |
| Cle-caspase3 | 9664S | Cell Signaling Technology | IHC(1:100） |
| *p*-H2AX | 9718S | Cell Signaling Technology | IHC(1:100） |

**Table S7: The sequences of primers and oligonucleotides used in qRT-PCR.**

| **Gene** | **Primer** | **Sequence (5'-3')** |
| --- | --- | --- |
| *CXCR4* | Forward | CACTTCAGATAACTACACCG |
|  | Reverse | ATCCAGACGCCAACATAGAC |
| *SOX2* | Forward | TGGACAGTTACGCGCACAT |
|  | Reverse | CGAGTAGGACATGCTGTAGGT |
| *NANOG* | Forward | TCTCCAACCATCCTGAACCTCAGCT |
|  | Reverse | GAGGCCTTCTGCGTCACACCA |
| *ALDH1A1* | Forward | CCACTCACTGAATCATGCCA |
|  | Reverse | TGAGCCAGTCACCTGTGTTC |
| *BMI-1* | Forward | CTGGTTGCCCATTGACAGC |
|  | Reverse | CAGAAAATGAATGCGAGCCA |
| *NOTCH* | Forward | GGTGAGACCTGCCTGAATG |
|  | Reverse | GTTGGGGTCCTGGCATC |
| *OCT4* | Forward | GCTGGAGCAAAACCCGGAGG |
|  | Reverse | TCGGCCTGTGTATATCCCAGGGTG |
| *KLF-4* | Forward | TACCAAGAGCTCATGCCACC |
|  | Reverse | CGCGTAATCACAAGTGTGGG |
| *β-CATENIN* | Forward | GAAACGGCTTTCAGTTGAGC |
|  | Reverse | TTCCATCATGGGGTCCATAC |
| *VIMENTIN* | Forward | AATGACCGCTTCGCCAAC |
|  | Reverse | CCGCATCTCCTCCTCGTAG |
| *β-ACTIN* | Forward | TACATGGCTGGGGTGTTGAA |
|  | Reverse | AAGAGAGGCATCCTCACCCT |

Below is the additional information related to the cell lines mentioned in the manuscript:

1. SKOV3 Cell Line

RRID: CVCL_0532.

Source: The SK-OV-3 cell line originates from human ovarian carcinoma and is commonly used to study ovarian cancer biology and drug sensitivity.

STR report: STR profiling revealed a concordant profile (EV: 0.9). No cross-contamination or human cell contamination was detected in this cell line. In this study, this cell line was used to induce drug resistance. According to the authentication criteria of the ATCC Standards Development Organization (ANSI/ATCC ASN-0002-2022), an EV match ≥ 80 % is considered evidence of relatedness and probable common ancestry.


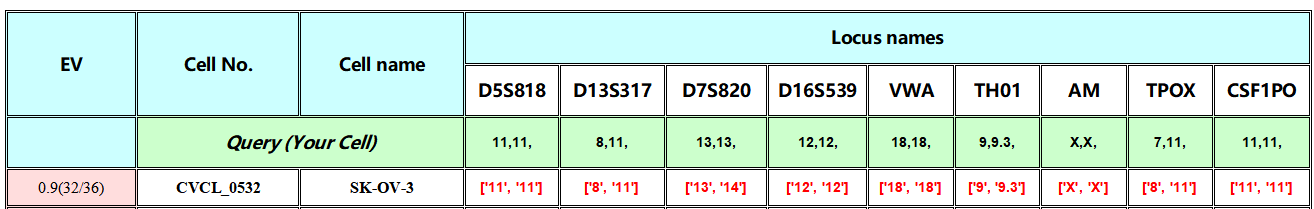


2. ES-2 Cell Line

RRID: CRL_1978.

Source: The ES-2 cell line is derived from human ovarian clear cell carcinoma and is widely used in cancer immunology and drug screening research.

STR report: STR profiling showed a complete match (EV: 1.0). No multiple alleles, cross-contamination, or human-cell contamination were detected in this cell line.


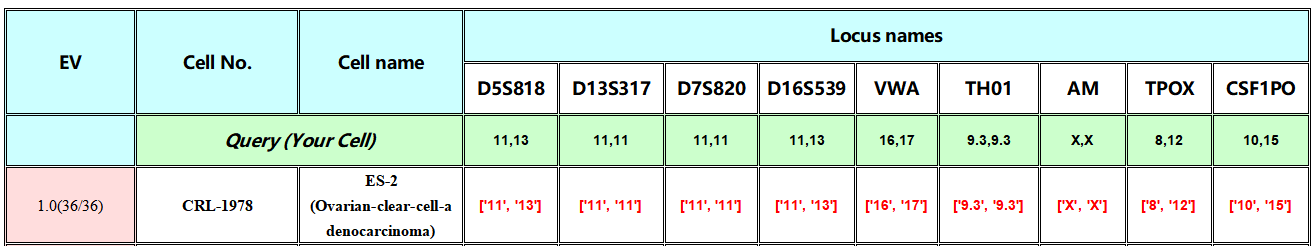


3. ID8 Cell Line

RRID: CVCL_IU14.

Source: The ID8 cell line is derived from murine ovarian epithelial cancer and is an important model used for studying ovarian cancer immunology.

STR report: STR profiling showed a complete match (EV: 1.0). No multiple alleles, cross-contamination, or human-cell contamination were detected in this cell line.

4 .Source of Purchase: All of the cell lines were obtained from the China Center for Type Culture Collection (Wuhan University, Wuhan, China).

5. Rationale for Using These Cell Lines:

These cell lines (SKOV3, ES-2, and ID8) are well-established and widely used cancer models with clearly defined sources and reliable supporting literature. SKOV3 and ES-2 are utilized in human ovarian cancer research, while ID8 is a crucial model for studying immune responses in ovarian cancer in mice. Therefore, the use of these cell lines is essential for the conclusions of our experiments and does not impact the reliability of the results.
